# High early-life gut Bacteroides links to microbiome stability, resilience, and risk for childhood infections

**DOI:** 10.1038/s41522-026-01022-7

**Published:** 2026-06-11

**Authors:** Matthias Hauptmann, Cornelia Gottschick, Uthayakumar Muthukumarasamy, Bianca Klee, Till Strowig, Rafael Mikolajczyk, Ulrich Emil Schaible

**Affiliations:** 1 Department of Cellular Microbiology, Research Center Borstel, Leibniz Lung Center, Borstel, Germany; 2 Institute for Medical Epidemiology, Biometrics and Informatics (IMEBI), Interdisciplinary Center for Health Sciences, Medical Faculty of the Martin Luther University Halle-Wittenberg, Halle (Saale), Germany; 3 Department of Microbial Immune Regulation, Helmholtz Center for Infection Research, Braunschweig, Germany; 4 Cluster of Excellence RESIST (EXC 2155), Hannover Medical School, Hannover, Germany; 5 Centre for Individualised Infection Medicine (CiiM), a joint venture between the Helmholtz-Centre for Infection Research (HZI) and the Hannover Medical School (MHH), Hannover, Germany; 6 German Center for Infection Research (DZIF), Partner site Hannover-Braunschweig, Braunschweig, Germany; 7 Helmholtz Center for Infection Research, Braunschweig, Germany; 8 Biochemical Microbiology and Immunochemistry, University of Lübeck, Lübeck, Germany; 9 German Center for Infection Research (DZIF), Partner Site Hamburg-Lübeck-Borstel-Riems, Borstel, Germany; 10 Leibniz Research Alliance INFECTIONS, Borstel, Germany

**Keywords:** Microbiota, Antimicrobials, Bacteria

## Abstract

Early childhood events, up to the age of two, are critical for the development of the microbiome and balanced immunity later in life. We investigated whether susceptibility to infections and microbiome resilience after antibiotic treatment are associated with key taxa in the early childhood microbiota. Therefore, we performed longitudinal microbiota analysis from stool samples of children within the German LoewenKIDS intensified subcohort. According to the exposure to antibiotic treatment, sample groups were defined as never-treated controls, 45–225 days pre-treatment (pre45-225), 0–30 days pre-treatment (pre0-30), 0–30 days post-treatment (post0-30), or >90 days post-treatment and age >540 days (post>90). 1176 stool samples of 162 children were included in our analysis, of which 49 children received antibiotics. Using generalized linear mixed models adjusted for age, we show that high abundance of *Bacteroides* was associated with receiving antibiotic treatment 45-225 days later, while low *Bacteroides* abundance before treatment was associated with low alpha diversity and increased beta diversity post treatment. Our data suggest a key role of the genus *Bacteroides* for the susceptibility to infections requiring antibiotic treatment and for microbiome stability and resilience in early childhood.

## Introduction

The human microbiome develops from early infancy to adulthood and is closely linked to the digestive and immunological functions of the host^1,2^. In the first weeks of life, the infant gut microbiome is colonized by pioneer bacteria, including *Lactobacillus*, *Enterobacter*, *Escherichia*, *Bacteroides*, *Parabacteroides*, and *Prevotella*, followed by a *Bifidobacteria-*dominated composition during breastfeeding. As the child grows, the microbiota gradually diversifies to a more complex composition, including a higher prevalence of Firmicutes and Bacteroidetes^2^. Many factors have been suggested to influence microbiome development during this critical phase of early human life, including delivery mode and early nutrition (i.e., breastfeeding vs. formula feeding)^3–8^. Vaginal delivery has been associated with an increased abundance of *Bacteroides* between 5 and 31 weeks of life. Additionally, the timing of breastfeeding cessation determined a reduction in the abundances of Bifidobacteria, Staphylococci, and Streptococci, along with an increased representation of Lachnospiraceae^9^. Significant shifts in the composition of the early childhood microbiome are completed by about 3 years of life. This time window is thus a critical phase that establishes the basis for health and well-being later in life^10,11^.

While in the past, severe bacterial infections such as tuberculosis, diphtheria, typhoid fever, and cholera contributed to high rates of early childhood mortality worldwide^12^, pediatric infectious diseases in industrialized countries today comprise infections of the ear, sinuses and other parts of the upper respiratory tract, and in most cases can either be cured with antibiotics or are caused by viruses^13^. Several studies have described associations between early childhood microbiome composition and childhood infections; however, more large-scale studies are needed to cover the dynamic changes in the early childhood microbiome linked to infections and antibiotic use^14^.

Antibiotic treatments are considered strong influencers of the microbiome both in adults and early childhood^15–17^, and use of antibiotics during early childhood has been linked to an increased risk of developing asthma, allergies, inflammatory bowel disease, and other inflammatory diseases later in life^14,18–22^. The mechanisms underlying these associations still need to be clarified. Early training of the immune system or long-term microbiome alterations are considered potential but non-exclusive reasons^23^. Increased awareness of the risks of antibiotic use, led to the reduction of outpatient antibiotic consumption in many industrialized countries, including Germany, between 2012 and 2021^24^, and a similar trend is also seen in pediatric antibiotic consumption^25^. Microbiome resilience describes the process of restoring the original microbiome composition and diversity upon perturbation-induced dysbiosis by influencers such as antibiotics. The time and degree of recovery have been analyzed for different antibiotic classes in adults^26^. However, few studies have investigated microbiome resilience in early childhood^27^. Our study addresses two aims. Aim 1 is to describe traits of the early childhood microbiome that are associated with susceptibility to childhood infections requiring antibiotic treatment. Aim 2 is to describe traits of the early childhood microbiome that are associated with microbiome stability and resilience upon antibiotic treatments.

## Results

### Study overview

**Fig. 1:**
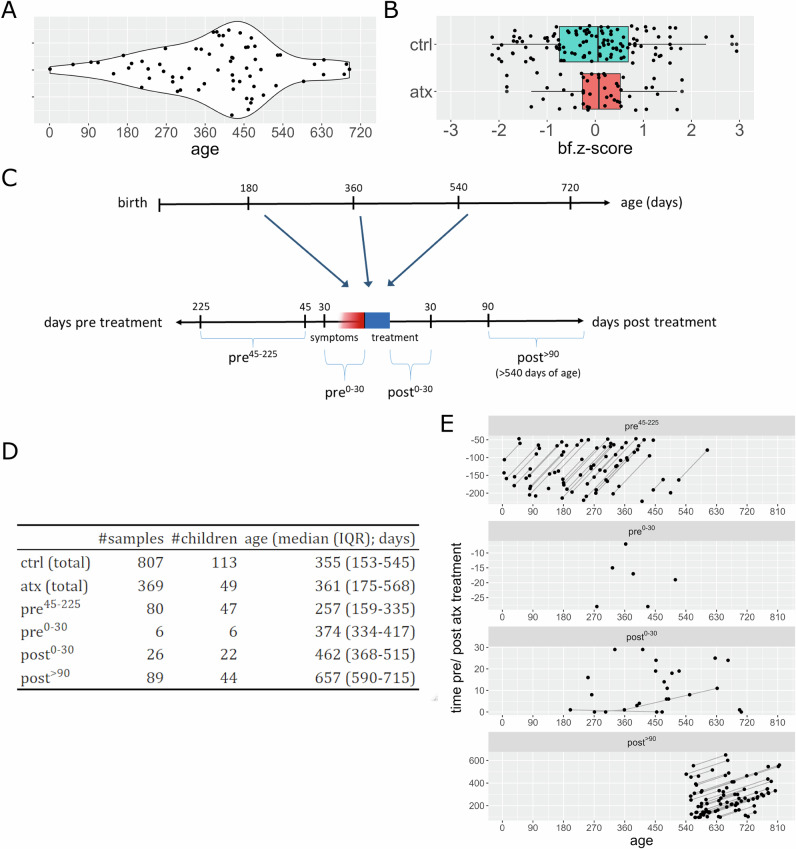
Analysis strategy. **A** Violin plot shows children’s ages at times of antibiotic treatment. **B** The plot shows the *z*-scores of the duration of breastfeeding (see “Methods” for details) of children in never-treated (ctrl) and antibiotic-treated (atx) groups. No significant difference was observed between the groups (Wilcoxon, *p* = 0.59). **C** The schematic shows the assignment of samples to observation phases before and after dysbiotic events used throughout this study. **D** Numbers of samples, numbers of children, and median (IQR) ages at times of sampling in the never-treated group (ctrl (total)), the antibiotic treatment group (atx (total)), and within the time frames defined in **C**. The numbers of children reflect those represented by at least one sample. **E** Dotplots depict age and time pre- or post-antibiotic treatment in the groups defined in **C**. Lines connect samples from identical children.

Two hundred eighty-six children were included in the intensified subcohort of the LoewenKIDS study. Of those, symptom diaries were not provided for 73 children. From 37 children, 16S sequencing was not completed and/or the symptom diaries were not completely collected and entered into the databases at the time of manuscript preparation, including data from 16 children who provided less than five regular stool samples. Four children were excluded because of antibiotic treatment before the age of 2 years, but after having provided the last routine stool sample. Two children were excluded due to conflicting data in the symptom diaries (see Supplementary Note 1). Eight children were excluded because less than five routine stool samples met the criterion of at least 15,000 16S sequencing reads per sample. Together, 1176 samples from 162 children were included in the analysis, among them 113 children who never received antibiotic treatment during the first 2 years of life and 49 children with at least one episode of antibiotic treatment (Table 1). Slightly more girls than boys were present among both children with or without antibiotic exposure. Among children who did not receive antibiotics, 80 (70.8%) were born by vaginal delivery and 31 (27.4%) by caesarean section (two not reported), while among antibiotic-exposed children, 40 (81.6%) were born by vaginal delivery, and nine (18.4%) by caesarean section (RR = 0.66, 95% CI [0.34, 1.28]). There was no difference in the duration of breastfeeding between children who received antibiotics in the first 2 years of life and those who did not (Fig. 1B). Thirty-nine children experienced one, six children experienced two, one child experienced three, and three children experienced four antibiotic treatment episodes. Aminopenicillins (36.4%) and cephalosporins (48.5%) were the most common antibiotic classes (others: 15.2%). The reasons for antibiotic treatment were ear, nose, and throat (ENT, 72.7%), gastrointestinal (GI, 1.5%), and urogenital (UG, 7.6%) infections (unknown/ other: 18.2%) (Table 1). Of the children in the antibiotic-exposed group, the median age when starting an antibiotic treatment episode was 417 days (IQR: 288–467 days; Fig. 1A). Sex ratios and delivery mode, as well as symptoms and type of applied antibiotics in the antibiotic treatment group of excluded children were comparable to those included in this study (Supplementary Table 1). From 113 children who did not receive antibiotic treatment in the first 2 years of life (never-treated controls), 807 samples were collected. From 49 children who received at least one antibiotic treatment episode, 369 samples were collected. Of those, 80 samples from 47 children were collected 45–225 days before an antibiotic treatment episode (pre^45-225^), six samples from six children were collected 0–30 days before an antibiotic treatment episode (pre^0–30^), 26 samples from 22 children were collected 0–30 days after an antibiotic treatment episode (post^0–30^), and 89 samples from 44 children were collected more than 90 days after an antibiotic treatment episode and at >540 days of age (post^>90^) (Fig. 1C–E). Even considering the short duration of the pre^0–30^ group, the number of samples in this group is unexpectedly low. A potential reason is that samples were considered as symptomatic when children were sick. In such cases, the regular samples have been skipped or provided later, and thus fell out of the pre^0–30^ time frame.

**Table 1:** Study overview

		Individuals			Antibiotic episodes by symptoms^b^				
		all	ctrl	atx	atx.all^a^	ENT	GI	UG	u/o
All		162	113	49	66	48	1	5	12
Sex									
	Male	74	50	24	35	26	1	0	8
	Female	88	63	25	31	22	0	5	4
Delivery mode^c^									
	Vaginal delivery	120	80	40	57	41	1	5	10
	Cesarean section	40	31	9	9	7	0	0	2
	Not reported	2	2	0	0	0	0	0	0
Antibiotic class									
	Aminopenicillin				24	20	0	1	3
	Cephalosporin				32	21	0	3	8
	Other				10	7	1	1	1

^a^39 children with 1, 6 children with 2, 1 child with 3, 3 children with 4 treatment episode(s).

^b^ENT=ear nose throat; GI=gastrointestinal; UG=urogenital; u/o=unknown/other.

^c^atx vs. ctrl, RR = 0.658 (CI: 0.339–1.275).

### Overview of microbiome composition

**Fig. 2:**
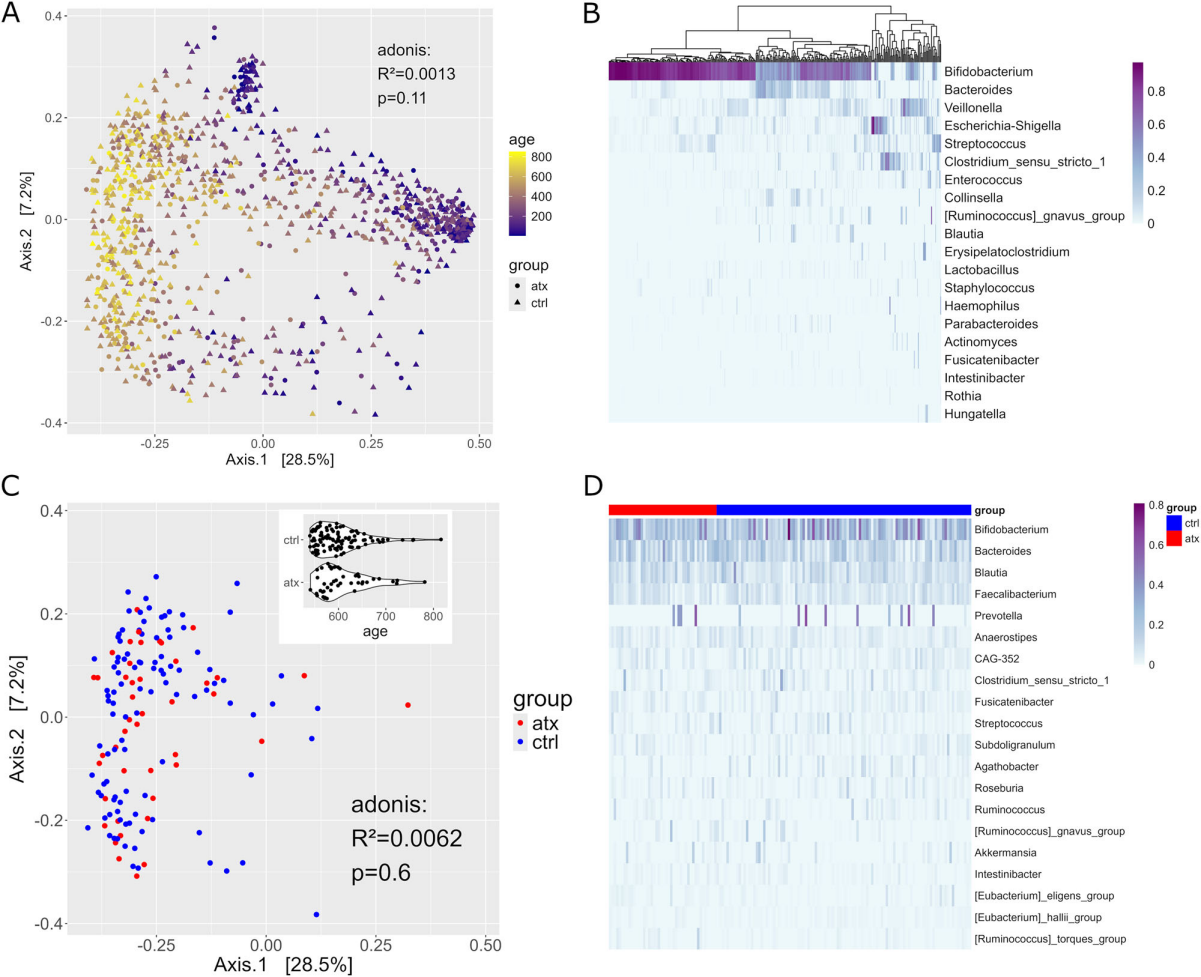
Microbiota composition by age. **A** PCoA of Bray-Curtis dissimilarities. Color represents the age of children; symbols represent the antibiotic-treated group (dots) vs. control group (triangles). Permutational ANOVA for the group effect revealed no significant difference between both groups (*p* = 0.11). **B** The heatmap shows relative abundances of the 20 most abundant genera at 0–180 days of age. **C** Same ordination as in **A**, but only samples in the late phase (i.e., age >540 days) and w/o antibiotic treatment during the last 90 days are displayed. The violin plot reveals comparable age distributions between antibiotic treatment and control groups. Permutational ANOVA for the group effect revealed no significant difference between both groups (*p* = 0.6). **D** The heatmap shows the relative abundances of the 20 most abundant genera for samples, as shown in **C**.

At 0–180 days of age, we found that the microbiomes of most children were dominated by *Bifidobacteria*. Some were characterized by a mix of *Bifidobacteria* and *Bacteroides*, and few were dominated by either *Escherichia/Shigella*, *Veillonella*, *Clostridium*, or *Streptococcus*, which corresponds to a loose clustering in a Principal Coordinate Analysis of Bray Curtis dissimilarities (PCoA) (Fig. 2 A, B). The microbiome compositions of children older than 540 days of age cluster more closely together in a PCoA plot. No differences were observed between the microbiome compositions of never-treated controls at >540 days of age and those of children who had received antibiotics and fall in the post^>90^ group of samples (permutational ANOVA, *p* = 0.6, Fig. 2C). While the microbiome at 540–851 days of age still contained high abundances of *Bifidobacteria*, the genera *Bacteroides*, *Blautia*, and *Faecalibacterium* were also prevalent and abundant. The prevalence of *Prevotella* was low. However, this genus reached high abundance in a subset of children, which was associated with low abundances of either *Bifidobacteria* or *Bacteroides*, or both (Fig. 2D).

### Microbiome alterations before and after antibiotic treatment (Aims 1 & 2)

**Fig. 3:**
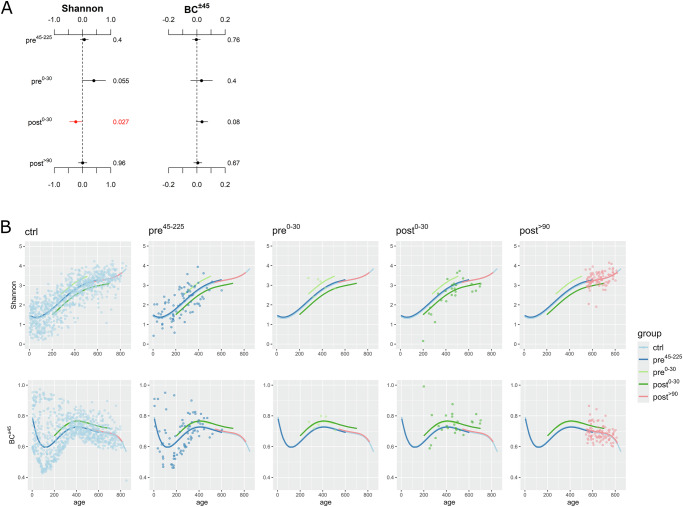
Microbiota α- and β-diversities by observation phase (Aims 1 & 2). Two linear mixed models for Shannon diversity or BC^±45^, respectively, have been fitted to the effects of age^†^, and observation phases (i.e., groups). Participant IDs were considered as a random factor. **A** Estimates with 95% confidence intervals and *p*-values for the effects of the groups are depicted. **B** Plots show actual Shannon diversities (top row) or BC^±45^ (bottom row) of individual samples by age and observation phase, as well as model-fits (lines, shown in all row plots for easier comparisons). Further model details can be found in Supplementary Table 2. ^†^ by 5th degree polynomials.

We explored the differences in microbiome diversity indices before and after antibiotic treatment episodes compared to never-treated controls. No Shannon index or BC^±45^ differences were observed between samples in the pre^45-225^ group and never-treated controls. Although not statistically significant, the Shannon index was higher in the pre^0–30^ group (likely coinciding with symptomatic or presymptomatic conditions) compared to never-treated controls (*β* = 0.40, 95% CI [−0.0086, 0.81], *p* = 0.055). At the same time, no apparent difference was seen in BC^±45^. In the post^0–30^ group, the Shannon index was reduced (*β* = -0.24, 95% CI [−0.45, −0.027], *p* = 0.027), and BC^±45^ was non-significantly increased (*β* = 0.036, 95% CI [−0.0043, 0.076], *p* = 0.08) compared to never-treated controls. No Shannon diversities or BC^±45^ differences were seen between samples in the post^>90^ group and never-treated controls (Fig. 3A, B).

**Fig. 4:**
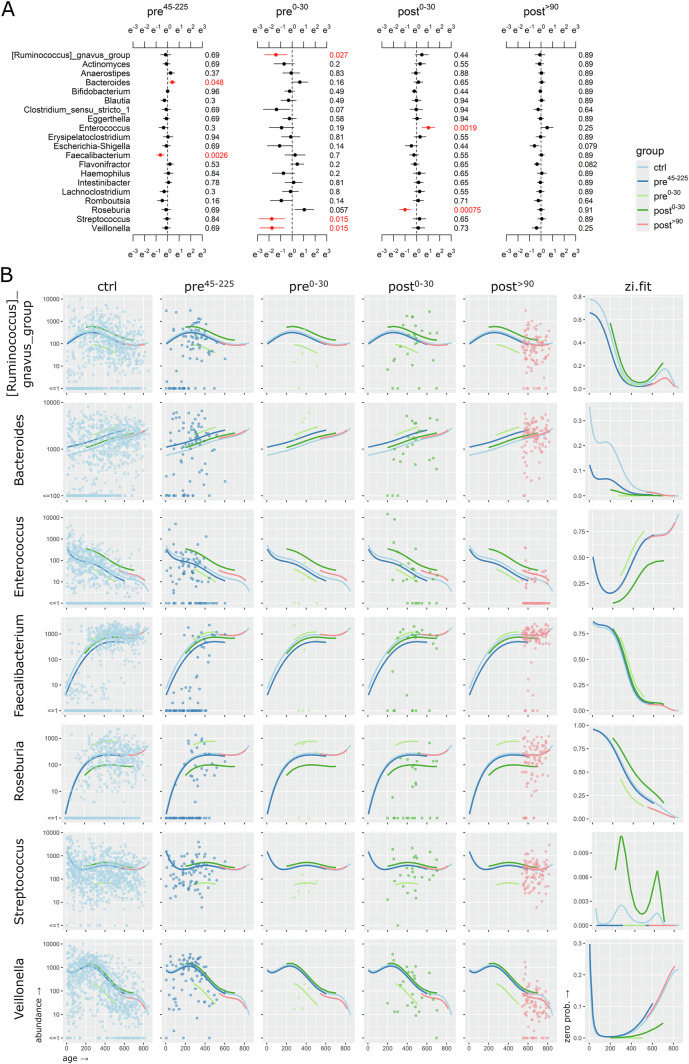
Differential abundances of genera by observation phase (Aims 1 & 2). Regression models for the abundance of 20 genera have been fitted to the effects of age^†^ and observation phases. Participant IDs were considered as a random factor. **A** Exponentiated estimates with 95% confidence intervals and *p*_adj_ of the observation phases are depicted. *p*_adj_ < 0.05 are highlighted in red. **B** Genera with significant estimates in at least one observation phase were selected. Plots show the abundances of individual samples by age and observation phase, as well as model fits (lines shown in all row plots for easier comparisons). Probabilities of excess-zeros (i.e., not explained by the neg. binomial part of the model), stratified by the observation phase, are shown in the rightmost plots. Further model details can be found in Supplementary Table 3. ^†^ by 5th degree polynomials. ^‡^ rarefied to 15,000 reads per sample.

We also explored differences in the abundances of the 20 most prevalent genera in the four groups (pre^45-225^, pre^0–30^, post^0–30^, post^>90^) compared to never-treated controls. We observed higher abundances of *Bacteroides* (e^β^ = 1.49, 95% CI [1.13, 1.97], *p*_adj_ = 0.048) and lower *Faecalibacteria* (e^β^ = 0.53, 95% CI [0.39, 0.73], *p*_adj_ = 0.0026) in the pre^45-225^ group. In the pre^0–30^ group, we found decreased abundances of the genera *[Ruminococcus]-gnavus group* (e^β^ = 0.25, 95% CI [0.094, 0.64], *p*_adj_ = 0.027), *Streptococcus* (e^β^ = 0.17, 95% CI [0.058, 0.51], *p*_adj_ = 0.015), and *Veillonella* (e^β^ = 0.17, 95% CI [0.061, 0.49], *p*_adj_ = 0.015). *Bacteroides* (e^β^ = 1.94, 95% CI [0.97, 3.86], *p*_adj_ = 0.16) and *Roseburia* (e^β^ = 2.84, 95% CI [1.27, 6.35], *p*_adj_ = 0.057), however, revealed increased abundances in the pre^0–30^ group when compared to the never-treated controls, though not in a significant manner. In the post^0–30^ group, the genus *Enterococcus* was increased (e^β^ = 2.79, 95% CI [1.64, 4.77], *p*_adj_ = 0.0019) and *Roseburia* was decreased (e^β^ = 0.37, 95% CI [0.23, 0.59], *p*_adj_ = 0.00075) compared to never-treated controls. Noteworthy, we found no significant alterations in the post^>90^ group compared to never-treated controls. However, a slightly increased presence of the genus *Enterococcus* remained in this time phase (Fig. 4A, B).

**Fig. 5:**
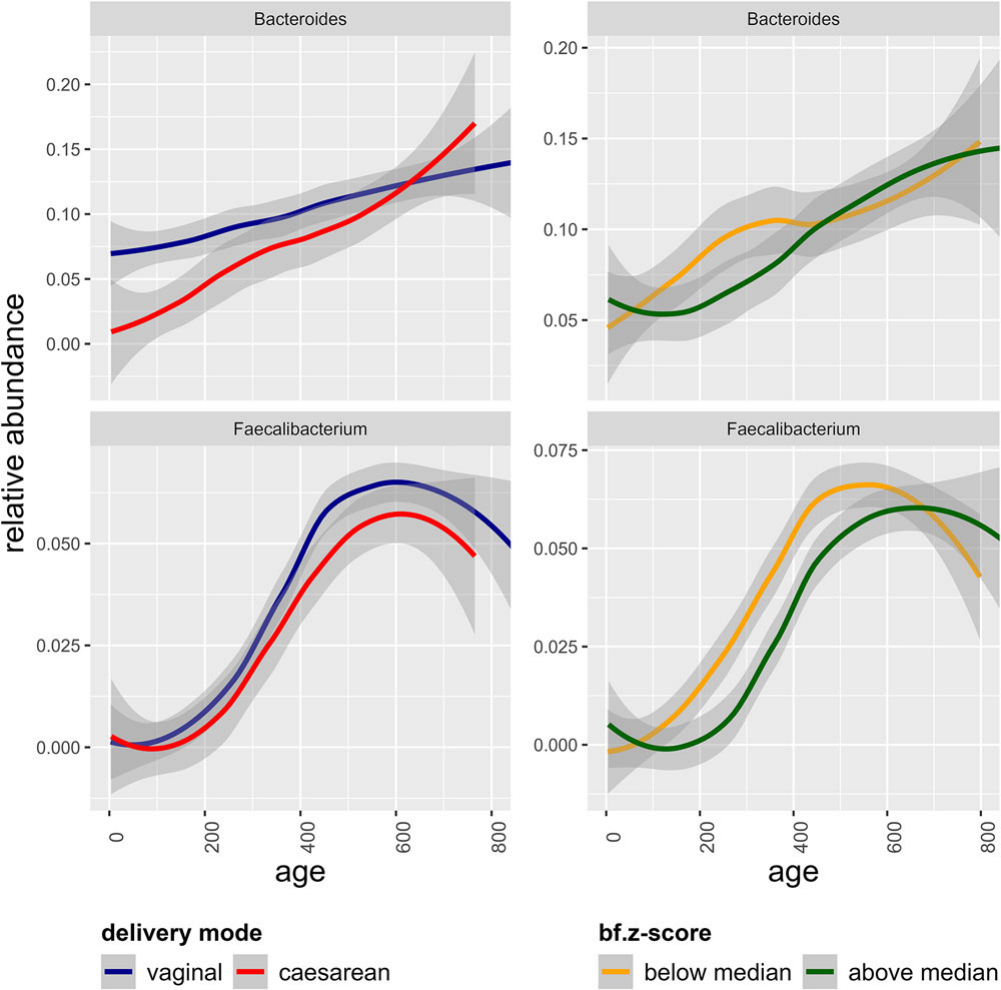
Effects of delivery mode and duration of breastfeeding (Aim 1). Relative abundances of *Bacteroides* or *Faecalibacterium* in the never-treated group were plotted against age. Lines show Loess-models for median values and 95% confidence intervals. Colors indicate delivery mode (vaginal delivery in blue, caesarian section delivery in red) or duration of breastfeeding (children with below median duration are shown in orange, children with above median duration are shown in green).

While altered taxa-abundances in the pre^0–30^ group were likely influenced by infections (the most frequent indication for antibiotics), we sought to further understand the altered abundances of *Bacteroides* and *Faecalibacterium* in the pre^45-225^ time frame. It is known that *Bacteroides* abundances vary between children born by vaginal delivery vs. caesarean section. Our data confirms a reduced *Bacteroides* abundance during the first year of life in children born via caesarean section vs. those born via vaginal delivery. This difference vanished during the second year (Fig. 5 and Table 2). Children with below-median duration of breastfeeding exhibited a slight and temporarily restricted increase in *Bacteroides* abundance compared to children with above-median duration of breastfeeding at about 0.5 to 1 year of age (Fig. 5). Nevertheless, after adjusting for the variables “delivery mode” and “duration of breastfeeding” (Table 2), a 52% increase in *Bacteroides* abundance was observed within the pre^45-225^ group compared to the never-treated controls (e^β^ = 1.52, 95% CI [1.05, 2.19], *p* = 0.027). In contrast, no interaction between ‘delivery mode’ or ‘duration of breastfeeding’ and allocation to the pre^45-225^ group was found (Table 2). This indicates that *Bacteroides* abundance at the time of sampling was higher in children who subsequently received antibiotic treatment within 45 and 225 days compared to children who remained antibiotic-free. Notably, this effect cannot be fully explained by “duration of breastfeeding” or “delivery mode”. *Faecalibacterium* abundance was independent of ‘delivery mode’ and even negatively associated with “duration of breastfeeding” (e^β^ = 0.83, 95% CI [0.73, 0.95], *p* = 0.0081) (Fig. 5 and Table 2). A 74.1% lower abundance of *Faecalibacterium* existed in the pre^45-225^ group compared to never-treated controls (e^β^ = 0.26, 95% CI [0.17, 0.40], *p* < 0.001), indicating that low *Faecalibacterium* abundance was associated with antibiotic treatment within the following 45–225 days. The strong positive interaction effect between the group factor (i.e., pre^45-225^ vs. never-treated controls) and delivery mode further indicates that this association is more robust in children born via vaginal delivery compared to children born via caesarean section (Table 2, and Supplementary Table 4).

**Table 2:** Effects of delivery mode and breastfeeding (Aim 1)

Dependent variable	Independent variable	e^β^	CI_low_	CI_high_	*p*-value
*Bacteroides* abundance	Group (pre^45-225^ vs. never-treated)	1.52	1.05	2.19	0.0274
	Delivery mode (C-section vs. vaginal)	0.661	0.475	0.921	0.0154
	Breastfeeding (*Z*-score)	0.95	0.826	1.09	0.48
	Group × Delivery mode interaction (pre^45-225^ & C-section)	0.819	0.369	1.82	0.625
	Group (pre^45-225^ vs. never-treated) × Breastfeeding interaction	0.859	0.591	1.25	0.427
*Faecalibacterium* abundance	Group (pre^45-225^ vs. never-treated)	0.259	0.167	0.403	1.5e-08
	Delivery mode (C-section vs. vaginal)	0.878	0.639	1.21	0.422
	Breastfeeding (*Z*-score)	0.832	0.727	0.952	0.00812
	Group × Delivery mode interaction (pre^45-225^ & C-section)	4.82	1.91	12.2	0.00108
	Group (pre^45-225^ vs. never-treated) × Breastfeeding interaction	0.661	0.409	1.07	0.0926

Multiple regression models were fitted for abundances of *Bacteroides* or *Faecalibacterium*, respectively, in a subset including only never-treated and pre^45-225^ samples (*n* = 846 samples from 152 children). The models simultaneously included the indicated independent variables, with age modeled using 5th-degree polynomials as fixed effects, and participant ID as a random effect. Model details are provided in Supplementary Table 4.

### Determination of microbiome resilience (Aim 2)

We explored factors that could influence microbiome stability and resilience after antibiotic treatment in early childhood using Shannon index and BC^±45^ in the post^>90^ group as indicators. No associations were found between Shannon index or BC^±45^ in post^>90^ samples with either of the factors “delivery mode”, “class of antibiotics”, “type of symptoms”, or ‘duration of breastfeeding’ (Table 3). Full models for Shannon index or BC^±45^, respectively, that included all factors revealed similar results (Supplementary Table 5).

**Table 3:** Factors that influence the degree of resilience (Aim 2)

Dependent variable	Independent variable		*β*	CI_low_	CI_high_	*p*-value
Shannon diversity	Delivery mode					
		Vaginal	-0.0652	-0.319	0.188	0.606
		Cesarean	0.255	-0.32	0.831	0.376
	Antibiotic class					
		Aminopenicillin	-0.097	-0.479	0.285	0.611
		Cephalosporin	0.123	-0.215	0.461	0.466
		Other	-0.329	-1.04	0.387	0.359
	Symptoms					
		Unknown/other	-0.191	-0.685	0.303	0.44
		ENT	0.0684	-0.2	0.337	0.609
		Gastrointestinal	-1.52	-3.02	-0.0284	0.046
		Urogenital	0.392	-0.798	1.58	0.51
	Breastfeeding					
		bf.z-score	0.00912	-0.236	0.255	0.941
Bray Curtis dissimilarity (BC^±45^)						
	Delivery mode					
		Vaginal	0.11	-0.168	0.387	0.43
		Cesarean	-0.34	-0.972	0.292	0.283
	Antibiotic class					
		Aminopenicillin	0.014	-0.413	0.441	0.948
		Cephalosporin	0.0196	-0.353	0.393	0.916
		Other	0.201	-0.591	0.993	0.611
	Symptoms					
		Unknown/other	-0.435	-0.966	0.095	0.105
		ENT	0.132	-0.159	0.423	0.364
		Gastrointestinal	1.36	-0.245	2.97	0.0946
		Urogenital	0.114	-1.14	1.37	0.855
	Breastfeeding					
		bf.z-score	0.0389	-0.225	0.303	0.768

14 children did not provide samples after day 540 and were excluded in this analysis.

A full model including all predictor variables has been fitted with similar results.

Model details are provided in Supplementary Table 5.

Two sets of four linear mixed-effects models were fitted separately, using Shannon diversity or BC^±45^ in the post^>90^ group as the dependent variable, as indicated in the first column. All models included age as a 5th-degree polynomial, participant as a random effect, and one of the factors “delivery mode”, “antibiotic class”, “symptoms”, or “breastfeeding”, as indicated in the second column. Statistical estimates and confidence intervals are shown for each variable coefficient.

**Fig. 6:**
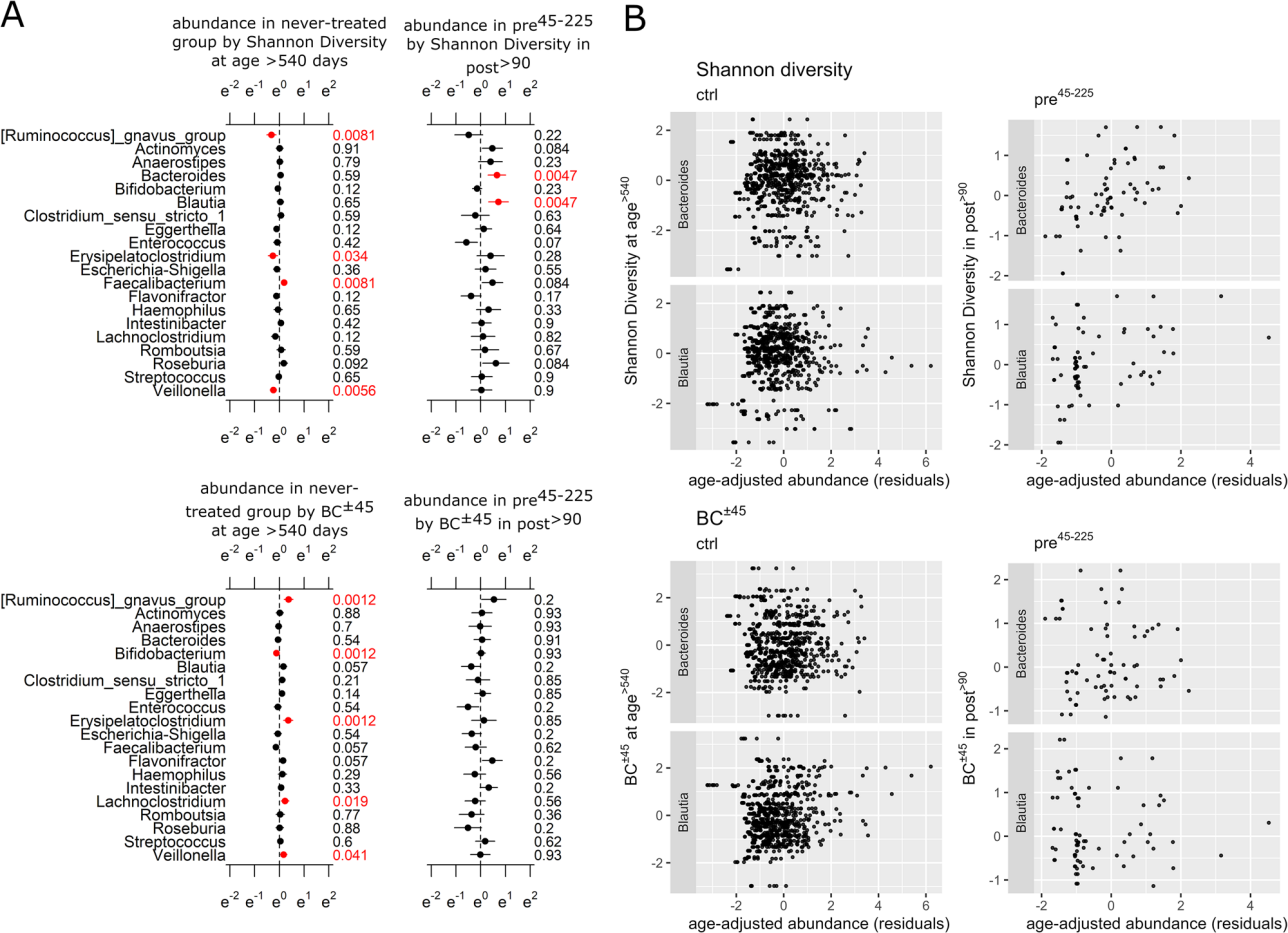
Identification of microbiota traits that are associated with the degree of resilience (Aim 2). **A** Regression models for the abundance of the 20 most prevalent genera in samples of the never-treated control group and the pre^45-225^ group have been fitted to the effects of age and the Shannon diversity (top panels) or BC^±45^ (bottom panel) in the post^>90^ group, stratified by the group factor. Participant IDs were considered as a random factor. Estimates, confidence intervals and adjusted *p*-values are depicted. **B** Reduced models were fitted by age^†^ and observation phase but not the Shannon or BC^±45^ in the post^>90^ group, respectively. Dot plots show the Shannon diversities (upper panels) or BC^±45^ (lower panels) in the post^>90^ groups by the residuals of the taxa abundances and thus serve as a visualization of the data presented in **A**. The selected genera show significant associations (p_adj_<0.05) between abundance in the pretreatment phase and Shannon diversity in the post^>90^ group as depicted in **A**. 14 children did not provide samples after day 540 of age and were excluded in the analysis. Further model details can be found in Supplementary Table 6. Additional models that control for the effects of age, delivery mode, and duration of breastfeeding in the never-treated control group are described in Supplementary Table 7. ^†^ by 5th degree polynomials.

To investigate whether patterns in the microbiome composition predicted its longterm stability or resilience after antibiotic treatment, we correlated relative abundances of the 20 most abundant bacterial genera in pre^45-225^ samples with the Shannon index and BC^±45^ in post^>90^ samples of the same children. Higher abundances of the genera *Bacteroides* (e^β^ = 1.92, 95% CI [1.35, 2.72], *p*_adj_ = 0.0047) and *Blautia* (e^β^ = 2.05, 95% CI [1.38, 3.03], *p*_adj_ = 0.0047) in pre^45-225^ samples were associated with higher Shannon diversity in post^>90^ samples (Fig. 6A, B, and Supplementary Table 6). No significant associations were found between abundances of any of the 20 most prevalent genera in the pre^45-225^ group and BC^±45^ in the post^>90^ group. In the never-treated control group, neither genera were associated with the Shannon index in the late phase (age >540 days), suggesting that *Bacteroides* and *Blautia* are not per se linked to a high Shannon index. However, the never-treated control group had a different age distribution than the pre^45-225^ group. To control for the possibility that *Bacteroides* or *Blautia*-abundances in a specific age range were associated with the Shannon index at >540 days of age, we stratified samples in the never-treated control group into four equally-sized age ranges (3–215, 215–427, 427–639, and 639–851 days of age). High *Blautia* abundances in the early age range (3–215 days) were associated with a higher Shannon index at the later time frame (Supplementary Table 7), while no such association was found for *Bacteroides*.

**Fig. 7:**
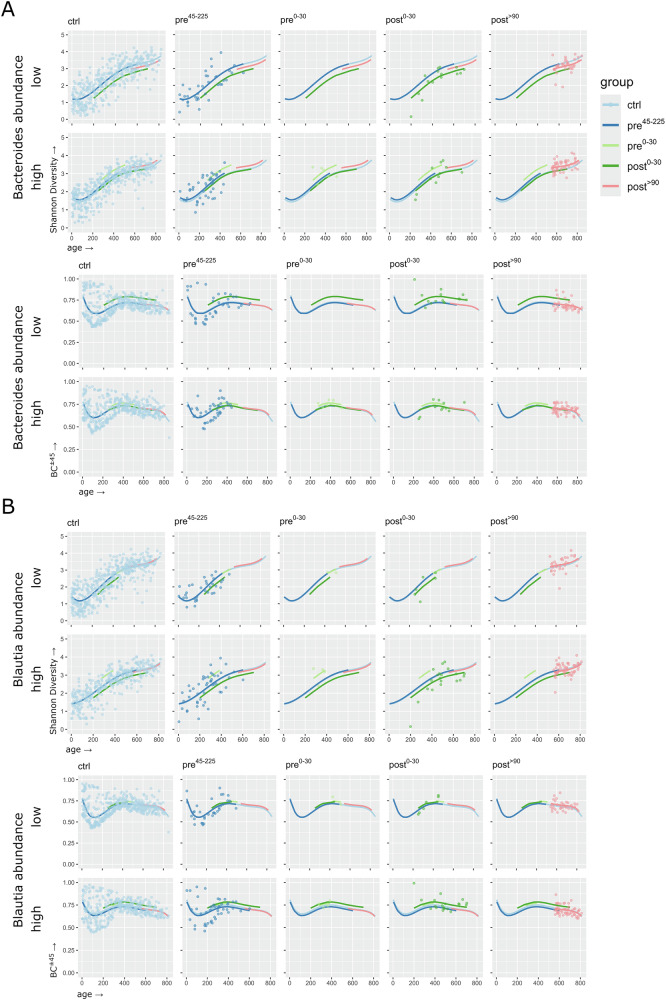
Microbiota resilience stratified by *Bacteroides* or *Blautia* abundances (Aim 2). Children were stratified into equal-sized groups based on the overall low- or high abundances (defined by random estimate residuals of models described in Fig. 4) of **A** *Bacteroides* or **B** *Blautia*. Shannon diversity and BC^±45^ were fitted to the effects of age^†^, and observation phases. Participant IDs were considered as a random factor. Plots show actual Shannon diversities of individual samples by age and observation phase, as well as model-fits (lines shown in all row plots for easier comparisons). Median (IQR) ages at times of sampling for the different sample groups are reported in the bottom-right corners of the Shannon-diversity plots. Further model details can be found in Supplementary Table 8. ^†^ by 5th degree polynomials.

While the above-described attempt was adjusted for age in the pre^45-225^ group, it was not possible to adjust for age in the post^>90^ group. We accepted this limitation, as the post^>90^ group was defined as >90 days post antibiotic treatment and age >540 days, where the influence of age on Shannon index and BC^±45^ is moderate (Fig. 3B). However, we were also interested in the effects of *Bacteroides* and *Blautia* abundances on microbiome stability and resilience shortly after the end of treatment (i.e., Shannon index and BC^±45^ in the post^0–30^ group of samples), where age has a stronger influence. Therefore, we stratified the dataset into children with low or high *Bacteroides* abundances across all samples from one child or similarly, into low or high *Blautia* abundances (see “Methods” for details). The median age at all sampling time points among all children with low *Bacteroides* abundance was 368 days (IQR: 168–566 days). Those children showed significantly decreased Shannon index (*β* = −0.42, 95% CI [−0.73, −0.10], *p* = 0.0094) as well as a significantly increased BC^±45^ (*β* = 0.065, 95% CI [0.0053, 0.13], *p* = 0.033) in the post^0–30^ group compared to controls, while no alterations in Shannon index or BC^±45^ were observed in children with high *Bacteroides* abundances (median age was 373 days (IQR: 182–578 days); Fig. 7A). The median age at all sampling time points among all children with high *Blautia* abundance was 389 days (IQR: 188–582 days). Although a significant decrease in Shannon index (*β* = −0.24, 95% CI [−0.47, −0.0025], *p* = 0.048) was seen in samples in the post^0–30^ group of children with high *Blautia* abundances, there was not a clear difference as compared to the children with low *Blautia* abundances (median age was 356 days (IQR: 162–565 days), reflecting the overall reduced Shannon index in post^0–30^ samples shown in Fig. 3.

Our observations indicate that low *Bacteroides* abundance in early childhood was associated with low α-diversity and high BC^±45^ after antibiotic treatment, which could not be explained by antibiotic-independent associations between *Bacteroides* abundance and α-diversity or BC^±45^.

### The role of *Prevotella* in microbiota-associated infection risk, stability, and resilience (Aims 1 & 2)

Since high abundances of *Bacteroides* have previously been associated with an industrial era lifestyle, we investigated the role of *Prevotella*, which has been reported to show a negative association with such a lifestyle. As the prevalence of *Prevotella* was low, which is consistent with previous reports, this genus failed the inclusion criterium for the 20 most prevalent genera. Therefore, it was not shown in the main results of Figs. 4–7. However, *Prevotella* reaches high abundances in non-negative samples (Fig. 2D, and Supplementary Fig. 1). To analyze differences in *Prevotella* abundances in the four treatment groups (pre^45-225^, pre^0–30^, post^0–30^, post^>90^) compared to never-treated controls, we applied a zero-inflated negative binomial model including treatment group as an independent variable. The pre^45-225^ group served as a proxy for future infections. We found a 64% lower relative abundance of *Prevotella* in the pre^45-225^ group and a 77% lower relative abundance in the pre^0–30^ group compared to never-treated controls (e^β^ = 0.36, 95% CI [0.15, 0.86], *p* = 0.022, and e^β^ = 0.23, 95% CI [0.07, 0.75], *p* = 0.015, respectively) (Supplementary Fig. 2).

To investigate the effects of *Prevotella* abundances on microbiome stability and resilience, we stratified the dataset into children with low or high *Prevotella* abundances across all samples from one child (see “Methods” for details). The median age at all sampling time points among all children with above-median *Prevotella* abundance was 353 days (IQR: 146–539 days). Among those, a lower Shannon index (*β* = −0.49, 95% CI [−0.80, −0.18], *p* = 0.0020) and a non-significant light increase in BC^±45^ (*β* = 0.048, 95% CI [−0.014, 0.11], *p* = 0.13) was observed in post^0–30^ samples compared to never-treated controls. In contrast, no differences were observed between post^0–30^ samples and never-treated controls among children with a below-median abundance of *Prevotella* (Supplementary Fig. 3).

## Discussion

In this longitudinal birth cohort study of 162 children followed up until the age of 2 years, we aimed to correlate the relative abundance of bacterial taxa within the early childhood microbiome and its overall state with the occurrence of childhood infections, microbiome stability, and resilience upon antibiotic treatment. We observed changes in microbiome compositions during early childhood and their effects upon treatment with beta-lactam antibiotics, as described before^9,28,29^. Within 30 days post antibiotic treatment, we observed a decrease in the Shannon index, increased *Enterococcus*, and decreased *Roseburia* abundances compared to never-treated controls, which is consistent with earlier reports^30–36^. Our data further show that these alterations are generally short-lived. We identified *Bacteroides* as a key genus associated with both the occurrence of childhood infections and increased stability and resilience of the gut microbiome upon antibiotic treatment. We also showed that high abundance of *Prevotella* was associated with low occurrence of childhood infections and low stability and resilience of the gut microbiome upon antibiotic treatment, indicating differential patterns of *Bacteroides*- vs. *Prevotella*-rich community types. We will further discuss these findings in the following paragraphs.

During the first 30 days after antibiotic treatment, we observed a reduced Shannon index, slightly but non-significantly increased β-diversity compared to never-treated controls, increased *Enterococcus*, and decreased *Roseburia* abundance. These results align with reports of reduced α-diversity during amoxicillin therapy^30^, altered community structure with increased *Enterococcus* after beta-lactam treatment^31,32^, and decreased *Roseburia* after therapies using aminopenicillins or cefprozil^33–35^. A review by Zimmermann et al. summarized the effects of different antibiotics in more detail^36^. Our data also indicate that these changes are generally short-lived. While few studies assess long-term beta-lactam effects, current literature mainly reports short-term reversible impacts, in contrast to longer-lasting effects from drugs such as clindamycin, ciprofloxacin, or macrolides^26,31,37,38^. Frequent early-life antibiotic use, especially in the early postnatal phase, has been associated with stronger microbiome alterations^16,17,39,40^. However, most children in our study were over 6 months of age at treatment, and our definition of long-term effects (≥90 days without antibiotics at >540 days of age) is strict, compared to the aforementioned studies. Thus, the limited long-term impact we observed aligns with previous reports.

Between 225 and 45 days before antibiotic treatment, we observed higher *Bacteroides* and lower *Faecalibacterium* abundances. As this period precedes treatment by several months, these patterns are unlikely to be driven by active infections and may instead reflect microbiome features that are associated with increased susceptibility to infections, which in most cases were ENT infections. We explored potential underlying factors: delivery mode and breastfeeding duration might relate to *Faecalibacterium*, but did not explain the *Bacteroides* associations. Still, other uncontrolled factors could explain them. Prior case-control studies linked *Bacteroides*-dominated microbiomes to higher bronchiolitis risk^41–43^ and report *Bacteroides* in mixed upper-respiratory infections^44–47^. Bacteroides-associated immunomodulatory functions may underlie these observations, including production of short-chain fatty acids, specifically succinate, which can inhibit neutrophil respiratory burst, an important defense strategy against extracellular bacterial pathogens that typically cause ENT infections^48^, and *Bacteroides* sphingolipid metabolism, which may modulate invariant natural killer T (iNKT) cells via CD1d^42,49,50^. To our knowledge, this is the first longitudinal study associating high intestinal *Bacteroides* abundance with later childhood respiratory or auditory infections. Future work should characterize *Bacteroides* strains through metagenomics to identify causal links.

We observed only mild, short-term microbiome changes after antibiotic treatment and no clear alterations in the post^>90^ group. To explore whether microbiome resilience depended on symptom type, antibiotic class, delivery mode, or breastfeeding duration, we analyzed each factor. Only gastrointestinal symptoms were associated with a lower late-phase Shannon index, though this is uncertain given that it was only a single case. Microbiome stability and recovery were generally higher in *Bacteroides*-rich communities, whereas *Bacteroides*-low communities recovered more slowly. *Bacteroides*-low microbiomes in early childhood typically exhibit lower α-diversity^39^. This supports our observation that *Bacteroides* abundance may mark communities more capable of post-antibiotic recovery. Mechanistic studies have shown that certain *Bacteroides* strains can stabilize the intestinal barrier or produce beta-lactamases that protect other taxa from antibiotics^51–55^. Chng et al. identified “recovery-associated bacteria” (RABs), including several *Bacteroides* species, which were enriched already before treatment and promote recovery via carbohydrate-active enzymes metabolizing mucus^56^. Mouse studies confirm that RABs can accelerate microbiome recovery and temporarily occupy niches for antibiotic-susceptible taxa^57–59^. This principle appears to be maintained across different perturbations, including beta-lactam antibiotics in children and azithromycin in cholera-infected adults, as reported by Hsiao et al.^59^. *Bifidobacterium longum* subsp. infantis, abundant during breastfeeding, similarly mitigates antibiotic-induced perturbations^60^. Collectively, these data suggest that microbiome stability and resilience depend on the presence of key taxa, including *Bacteroides* and related RABs, adapted to host nutrient sources such as human milk oligosaccharides in early life and mucus thereafter.

In our study, high abundance of the genus *Bacteroides* was the strongest and statistically most robust predictor of a stable and resilient microbiome and increased susceptibility to childhood infections, in most cases of the ENT system. In our study, this “high-*Bacteroides”* community-type is further characterized by high α-diversity, abundance of *Blautia* and low prevalence and abundance of *Prevotella*. Western lifestyle, including ingestion of high-calorie/low-fiber food, indoor stay, hygienic environment, reduced physical activity, reduced exposure to sunlight, and medical interventions, led to an adaptation of the microbiome, which became stable due to transgenerational inheritance^61^. The most striking adaptations include a rise in *Bacteroides* abundance and a loss of *Prevotella* prevalence and abundance. Some studies also reported an increased abundance of *Blautia* and *Ruminococcus*. On the one hand, high *Bacteroides* abundance promoting childhood infections could have been of evolutionary disadvantage in the past, while nowadays, these infections have lost much of their former threat due to better health, nutritional and hygiene standards, vaccination and the availability of antibiotic therapies. On the other hand, high frequency of dysbiotic events, including those caused by antibiotics, relays on the stabilizing effect of *Bacteroides* for a Western-type microbiome.

The strengths of this study are the frequent stool sampling combined with comprehensive metadata in a well-defined population of children. At the same time, the relatively small case number and the use of 16S rRNA amplicon instead of shotgun metagenomic sequencing are limitations. Although a considerable number of children were excluded from the analyzes, sex, delivery mode, frequencies and types of antibiotic treatment, as well as symptoms, were comparable between those children included and the excluded ones for which symptom diaries were available. Data about the indications for antibiotic use had to be reconstructed based on reports from the parents, which in most cases were based on a “diagnosis given by a physician” on the day of the doctoral visit. Since no data about the causative pathogens were available, it is possible that in some cases, antibiotic treatment was not necessary. Potentially, this limited the statistical power of associations between microbiota composition and later infections. Childhood infections that did not require antibiotic treatment were not considered in this study and therefore exist in both the never-treated control group and all observation phases in the antibiotic-treatment group and likely contribute to the unexplained variability in the data. The peculiar distribution of *Prevotella* is not perfectly reflected by a negative binomial model, even when including a zero-inflation part, and the statistical power of a logistic model was too low with the sample size investigated here (data not shown). Therefore, the results for *Prevotella*, though interesting to follow up, have to be interpreted with caution.

Our data reveal that *Bacteroides* is important for the stability and resilience of the early childhood microbiome and is associated with higher susceptibility to childhood infections.

## Methods

### Study design

The LoewenKIDS study is an observational birth cohort from five study regions in Germany (Braunschweig, Hannover, Halle (Saale), Munich, and Bremen), which has been described in detail^62^. The study has been registered at Clinicaltrials.gov (Identifier: NCT02654210). In brief, participants (*n* = 782) were recruited from 2015 to 2018 and have been observed from birth onwards with an intensive study protocol in the first 6 years of life. Parents kept a daily symptom diary on respiratory and gastrointestinal symptoms and daily information on medication, such as antibiotics. Questionnaires were provided to the parents at the age of 6 months, 1 year, and once a year afterwards. This study was restricted to data from children out of a subcohort (*n* = 286), in which parents collected routine stool samples four times per year during the first 2 years of life. Parents additionally collected symptomatic stool samples whenever the child had gastrointestinal symptoms (diarrhea or vomiting), but symptomatic samples were excluded in the current analysis. Participants with less than five routine stool samples after quality filtering and rarefaction of the sequencing data, without accessible symptom diaries, with antibiotic treatment after having provided the last routine stool sample, or with contradictory information on antibiotic treatment in the symptom diaries were excluded (see details in Supplementary Note 1 as well as in “Results: study overview”). Samples of children who did not receive antibiotics in the first 2 years of life were classified as never-treated controls. Samples of children who received antibiotic treatment at least once during the first 2 years of life were divided into four groups, according to the temporal relationship to antibiotic treatment episodes: 45–225 days prior to the first-ever dose of antibiotics (pre^45-225^), 0–30 prior to the first-ever dose of antibiotics (pre^0–30^), 0–30 days following completion of an antibiotic treatment episode (post^0–30^), or >90 days without any antibiotic treatment and >540 days of age. The latter represents an age range where the microbiome composition is relatively stable (post^>90^).

### Data extraction from questionnaires and symptom diaries

Information about the delivery mode was used as reported by the participants. Questionnaires contained three items on the duration of breastfeeding (“any breastfeeding”, “mostly breastfeeding”, and “exclusive breastfeeding”) in months. To combine the three items into a single metric, their values were first individually scaled using the root mean square method to harmonize the different value ranges. Of those, means were calculated so that all items contributed equally to the data. Finally, *z*-scores were calculated, which are reported as the combined breastfeeding value (bf.z-score).

Parents were asked to report medications in the free-text columns of the symptom diary. Either the active agents or tradenames were reported. We extracted the data on antibiotic drugs and classified them as aminopenicillins, cephalosporins, and ‘others’. Intervals in which children received daily antibiotic treatments were defined as antibiotic treatment episodes. Details regarding episodes where treatment was temporarily interrupted, or the type of antibiotics was changed, are reported in Supplementary Note 1.

In the symptom diaries, parents were asked to provide reasons for a visit to a physician and the diagnosis given by a physician in free-text fields. Reasons for antibiotic treatment (which requires a medical prescription in Germany) were extracted from symptom diaries, which we screened in a time frame of ten days preceding antibiotic treatments. Our first criterion was the diagnosis, followed by the reasons for a visit to a physician. When free-text information was not available, we classified reasons based on the reported symptoms into “ear, nose, and throat” (ENT) infection, “gastrointestinal” (GI) infection, or “unknown/ other”. Items for ENT symptoms were “wheezing”, “runny or stuffy nose”, “shivering”, and “throat pain”, each asking for scores from none (0) to severe (3), as well as the item “cough”, asking for the categories “dry”, “with expectoration”, or “unknown”. Items for GI symptoms asked for frequency of vomiting and observation of ‘mushy/ liquid stools’.

### 16S rRNA gene sequencing

Samples in RNAsepar (Biosepar, Simbach, Germany) were sent by the parents through postal mail to the Helmholtz-Centre for Infection Research in Braunschweig (Germany), where they were aliquoted and stored at -80°C until further analysis. DNA was extracted from samples using a DNA extraction kit (ZymoBIOMICS 96 MagBead DNA Kit, Zymo Research, Irvine, CA, US). Subsequently, the V4 region of the 16S rRNA genes was amplified in one step using primers targeting the 515F–806R region as described by Caporaso et al.^63^. The primers contained Illumina adapter-, pad-, and linker sequences, and the reverse primers additionally contained Golay barcodes. The amplicons were sequenced using a v2 (2 ×250 bp) chemistry on an Illumina MiSeq instrument. Sequences were demultiplexed, paired reads were merged, trimmed (fw reads 6–250 bp, rv reads to 6-180 bp), and quality filtered (max. expected errors = 3). The resulting sequences were denoised and chimera-filtered to obtain amplicon sequence variants using QIIME2 with DADA2 version 2021.8.0^64,65^.

### Data analysis

Data were analyzed using R Studio with R version 4.2.2. Relative risks were calculated using the epitools package (version 0.5-10.1). Microbiome data were analyzed using phyloseq version 1.40.0. Data were rarefied to 15,000 reads per sample. Data from children with less than five samples of sufficient quality were excluded from further analysis. Shannon diversities and Bray–Curtis dissimilarities (BC) were calculated using the vegan package (version 2.6-2). Mean BC compared to age-matched never-treated controls was calculated for each sample by taking the arithmetic mean of all BC between a sample and all samples of the never-treated control group that fall into an age range between 45 days younger or older (BC^±45^). Permutational ANOVA was calculated with the function adonis2, also using the vegan package (version 2.6-2). Heatmaps were made using the pheatmaps package (version 1.0.12).

Linear mixed-effects models using the restricted log-likelihood method (function lme of nlme package version 3.1-153) were used to analyze Shannon diversities and BC^±45^. Age was included as fixed factor using 5th degree polynomials, and participant ID was included as a random factor. We report β-coefficients, which describe the estimated differences in absolute values for Shannon diversity or BC^±45^, respectively. The 20 most prevalent taxa (used in Figs. 4 and 6) were determined among all samples (incl. the entire age range). Zero-inflated negative binomial mixed models were fitted using the package NBZIMM 1.0^66^, accounting for the fact that microbiome sequencing data are zero-inflated due to a combination of below detection limit abundances and absolute physical absence of taxa in the original habitat (i.e., the gut). For Shannon index and BC^±45^, age was included as a fixed effect using 5th degree polynomials, and the participant ID was included as a random factor. In non-converging models, 4th degree polynomials of age were used (detailed in Supplementary Tables). We report exponentially transformed β-coefficients (e^β^) for these models, which describe the estimated fold-changes in taxa abundances. For illustrations in Fig. 4 and Supplementary Fig. 2, abundances were transformed by log_10_(count+1). False discovery rate (fdr-) adjustment was used to correct for the comparisons of multiple taxa (adjusted values are indicated as p_adj_). For Fig. 5, Loess models were fitted using the stats package (version 4.2.2). To define microbiome subsets with low vs. high abundance of a given genus (for Fig. 7 and Supplementary Fig. 3), random effect residuals were extracted from models as described in Fig. 4 (*Bacteroides* and *Blautia*) and Supplementary Fig. 2 (*Prevotella*). These represent the degree by which a taxon in a child *participating in the study* generally differs from the overall model fit, i.e., over the entire age range and under consideration of the grouping factors for the individual samples. Children with below-median residuals of a given taxon were grouped in the low-abundance subset, whereas those with above-median residuals were grouped in the high-abundance subset.

Marginal and conditional *R*^2^ values were calculated for all models using the command r2_nakagawa from the performance package (version 0.9.2)^67^. In some cases, marginal and conditional *R*^2^ could not be estimated because of groups with no values, and no information was given in these cases (Supplementary Table 7). Confidence intervals of all models were calculated using the nlme package (version 3.1-153).

### **Ethics approval and consent to participate**

The Ethics Committees of the Martin Luther-University Halle-Wittenberg (No. 2016-04), the Medical School Hannover (No. 6794), and the Ludwig Maximilian University Munich (No. 445−15), Germany approved the study. Parents received detailed information on the objectives of the cohort study and provided written informed consent.

## Supplementary information

Supplementary Information

Supplementary data 1

## Data Availability

The dataset supporting the conclusions of this article is available at EMBL’s European Bioinformatics Institute under accession number PRJEB79757 (https://www.ebi.ac.uk/ena/browser/view/PRJEB79757). The metadata are available in Supplementary Data 1.
