# Supplementary material for: High early-life gut Bacteroides links to microbiome stability, resilience, and risk for childhood infections
Source: NPJ Biofilms Microbiomes. 2026 Jun 11;12:115. doi: 10.1038/s41522-026-01022-7 (PMC13254207; doi:10.1038/s41522-026-01022-7)
Supplement: Supplementary file 1 — Supplementary Information [file 41522_2026_1022_MOESM1_ESM.docx]

Supplementary Notes

**Supplementary Note 1:**

**Cases that were excluded due to controversial data in the symptom diaries:**

2 cases were excluded due to controversial data in the symptom diaries. Of those, one case had an incomplete symptom diary, likely because the data were not transferred completely into the database. For the other case, application of "Penicillin-Ratiopharm 125mg Suppositories" was reported. Likely Paracetamol was given.

**Treatment episodes with treatment breaks or changes of types of antibiotics:**

Five cases received treatment episodes that were paused for 1 day (2 cases), 2 days (1 case), 3 days (1 case), or 5 days (1 case). Each of those was counted as one episode.

One case received various types of antibiotics (Aminopenicillins, Cephalosporins, Aminoglycosides) for 25 days, with breaks of max. 4 days in between. This was counted as one episode with antibiotic class "other".

One case received intravenous antibiotics (antibiotic class unknown) for one day, followed by oral Cefpodoxim treatment. This was counted as one episode of Cephalosporin treatment.

One case received antibiotics for 13 days, and the type of antibiotic was only specified on days 10-13 as Amoxiclav. This was counted as one episode of Aminopenicillin treatment.

One case received Cefpodoxim for 11 days and Amoxicillin for 14 days, starting from the 5th day of Cefpodoxim (resulting in 18 days treatment in total). This was counted as one episode with antibiotic class "other".

One case received Cefaclor for 3 days and Amoxicillin for 8 days, starting from the 3rd day of Cefaclor (resulting in 10 days treatment in total). This was counted as one episode of Aminopenicillin treatment.

One case received Cefuroxim for 1 day, followed by 14 days of Cotrimoxazol. This was counted as one episode with antibiotic class "other".

One case received Kanamycin POS eye drops. Since this was a topical treatment, it was not counted as antibiotic treatment episode for this study.

Supplementary Figures


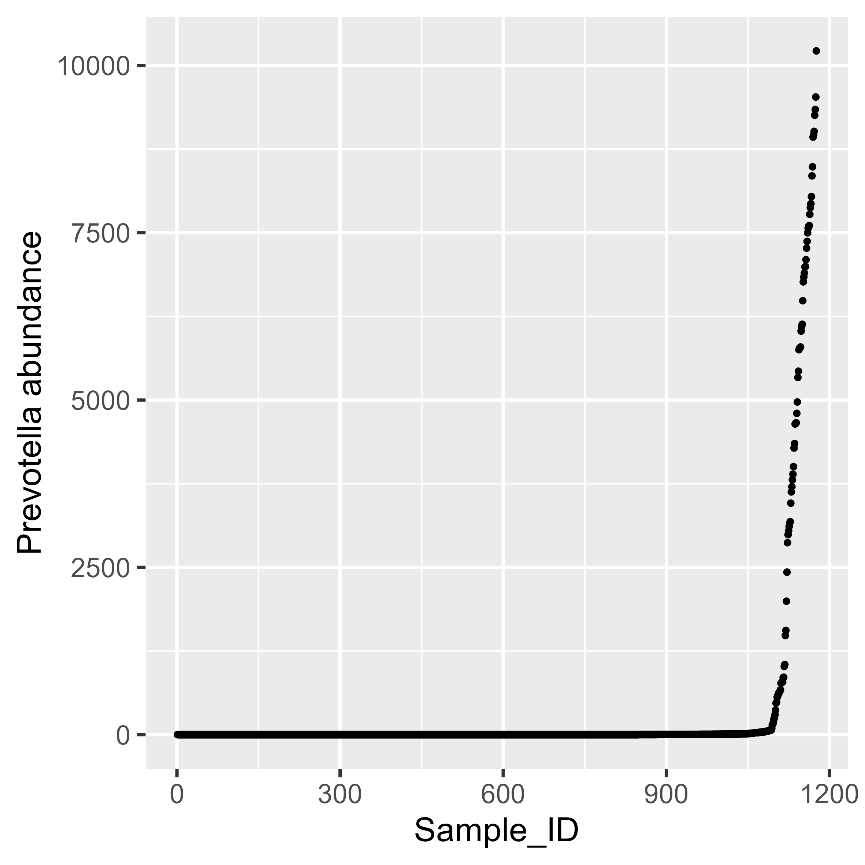


**Supplementary Figure 1:** Graph shows the *Prevotella* abundances of all samples, in increasing order. (Data have been rarefied to 15,000 reads per sample.)

**Supplementary Figure 2: Differential abundances of *Prevotella* by group.** Regression models for the *Prevotella* abundance have been fitted to the effects of age^†^ and group. Participant IDs were considered as a random factor. **A)** Plots show abundances^‡^ of individual samples by age and group, as well as model-fits (lines, shown in all row plots for easier comparisons). Probabilities of excess-zeros (i.e. not explained by the neg. binomial part of the model), stratified by group, are shown in the rightmost plots. **B)** Model details and statistics.


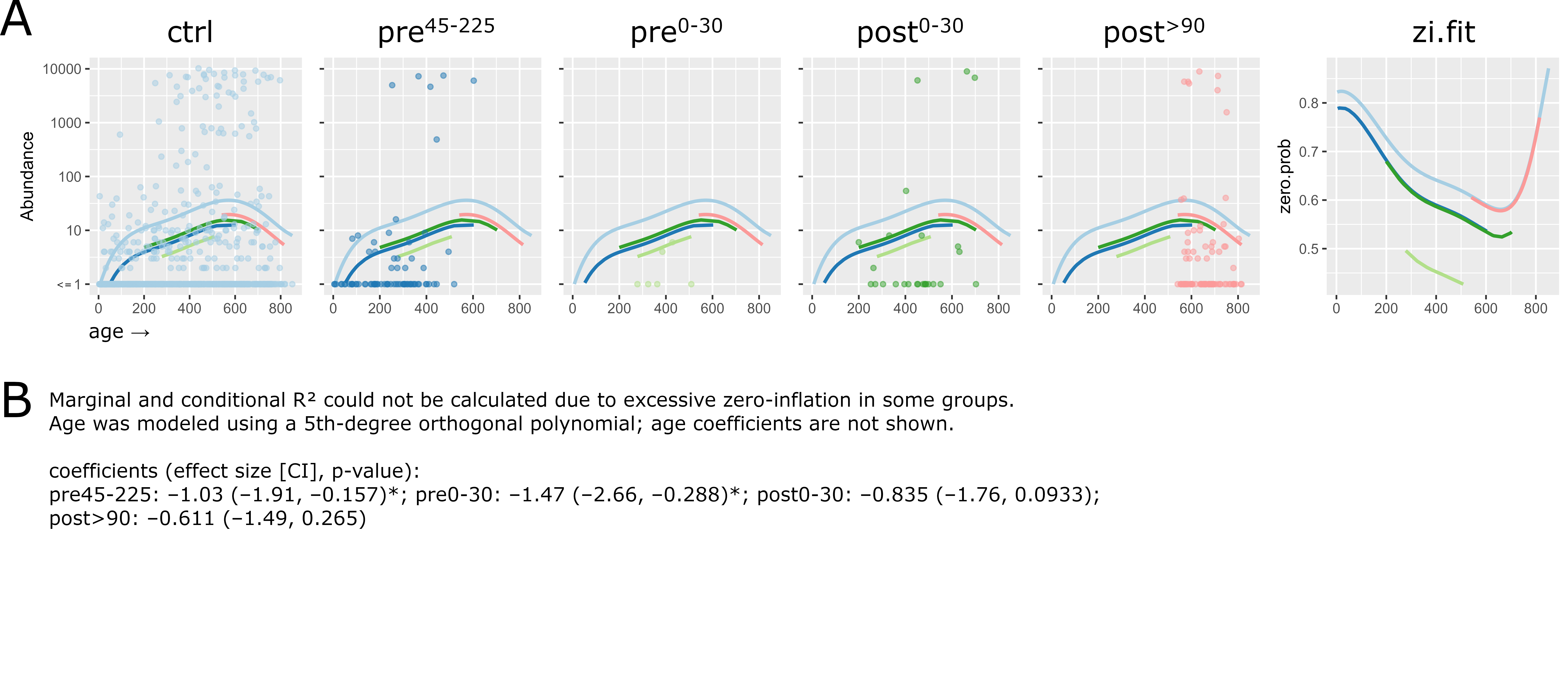


^†^ by 5^th^ degree polynomials. ^‡^ rarefied to 15,000 reads per sample.





**Supplementary Figure 3: Microbiota resilience stratified by *Prevotella* abundance.** Children were stratified into equal-sized groups based on the overall low- or high abundances (defined by random estimate residuals of models described in Fig. S2) of *Prevotella*. Shannon diversity and BC^±45^ were fitted to the effects of age^†^ and observation phases. Participant IDs were considered as a random factor. **A)** Plots show actual Shannon diversities of individual samples by age and group, as well as model fits (lines shown in all row plots for easier comparisons). Median (IQR) ages at times of sampling for the different sample groups are reported in the bottom-right corners of the Shannon-diversity plots. **B)** Model details and statistics.

^†^ by 5^th^ degree polynomials.

Supplementary Tables

**Supplementary Table 1. Overview of excluded participants**

|  |  | **individuals** | | | **antibiotic episodes by symptoms^1^** | | | | |
| --- | --- | --- | --- | --- | --- | --- | --- | --- | --- |
|  |  | all | ctrl | atx | atx.all | ENT | GI | UG | u/o |
| **all** |  | 51 | 36 | 15 | 38 | 32 | 1 | 1 | 4 |
| **sex** |  |  |  |  |  |  |  |  |  |
|  | male | 25 | 17 | 8 | 28 | 24 | 1 | 1 | 2 |
|  | female | 26 | 19 | 7 | 10 | 8 | 0 | 0 | 2 |
| **delivery mode** |  |  |  |  |  |  |  |  |  |
|  | vaginal delivery | 36 | 24 | 12 | 31 | 26 | 1 | 1 | 3 |
|  | cesarean section | 12 | 9 | 3 | 6 | 5 | 0 | 0 | 1 |
|  | not reported | 3 | 3 | 0 | 1 | 1 | 0 | 0 | 0 |
| **antibiotic class** |  |  |  |  |  |  |  |  |  |
|  | Aminopenicillin |  |  |  | 16 | 13 | 0 | 0 | 3 |
|  | Cephalosporin |  |  |  | 12 | 10 | 1 | 1 | 0 |
|  | other |  |  |  | 10 | 9 | 0 | 0 | 1 |

^1^ ENT=ear nose throat; GI=gastrointestinal; UG=urogenital; u/o=unknown/other

### Supplementary Table 2. Summary of Regression Models used in Figure 3.

Linear mixed regression models were fitted to Shannon diversity or Bray-Curtis dissimilarity (BC) with participant IDs as a random factor and age modeled as a 5th-degree orthogonal polynomial (coefficients not shown). Marginal and conditional R² values are reported for each model. Group effects are presented for pre45-225, pre0-30, post0-30, and post>90 with estimates, confidence intervals, and significance levels indicated by asterisks.

| **DV** | **Model** | **Marginal R²** | **Conditional R²** | **Group effects (effect size [CI], p-value)** |
| --- | --- | --- | --- | --- |
| Shannon | reduced | 0.671 | 0.753 | — |
| Shannon | full | 0.674 | 0.756 | pre45-225: 0.0614 [-0.0812, 0.204]; pre0-30: 0.398 [-0.00863, 0.805]; post0-30: -0.241 [-0.454, -0.0274]*; post>90: 0.00398 [-0.144, 0.152] |
| Bray-Curtis | reduced | 0.162 | 0.404 | — |
| Bray-Curtis | full | 0.165 | 0.407 | pre45-225: -0.00423 [-0.0315, 0.023]; pre0-30: 0.0327 [-0.0428, 0.108]; post0-30: 0.0358 [-0.00427, 0.0758]; post>90: 0.00606 [-0.0223, 0.0344] |

**Notes:**

- Reduced models do not include group effects (marked “–”).
- DV = dependent variable
- CI = 95% confidence interval; * = p<0.05

### Supplementary Table 3. Summary of Regression Models used in Fig. 4.

Zero-inflated negative binomial mixed-effects models were fitted for each taxon, with age modeled using orthogonal polynomials (4th- or 5th-degree, depending on model convergence) and a random intercept for participant ID in reduced and full models; full models additionally included group effects (pre/post exposure windows). Group effects are presented for pre45-225, pre0-30, post0-30, and post>90 with estimates, confidence intervals, and non-adjusted significance levels indicated by asterisks; age polynomial coefficients are not shown.

| **Taxon** | **Model** | **Age poly degree** | **Marg. R²** | **Cond. R²** | **Group effects (estimate [95% CI], significance)** |
| --- | --- | --- | --- | --- | --- |
| **[Ruminococcus]_gnavus_group** | reduced | 5 | 0.126 | 0.655 | – |
|  | full | 5 | 0.138 | 0.661 | pre45-225: -0.17 [-0.61, 0.26]; pre0-30: -1.41 [-2.36, -0.45] **; post0-30: 0.45 [-0.10, 1.00]; post>90: -0.10 [-0.52, 0.33] |
| **Actinomyces** | reduced | 5 | 0.298 | 0.552 | – |
|  | full | 5 | 0.300 | 0.562 | pre45-225: -0.10 [-0.41, 0.21]; pre0-30: -0.70 [-1.56, 0.16]; post0-30: 0.31 [-0.15, 0.76]; post>90: -0.13 [-0.48, 0.23] |
| **Anaerostipes** | reduced | 5 | 0.696 | 0.804 | – |
|  | full | 5 | 0.692 | 0.802 | pre45-225: 0.26 [-0.07, 0.59]; pre0-30: -0.08 [-0.80, 0.64]; post0-30: -0.06 [-0.44, 0.32]; post>90: 0.03 [-0.24, 0.30] |
| **Bacteroides** | reduced | 5 | 0.099 | 0.481 | – |
|  | full | 5 | 0.124 | 0.485 | pre45-225: 0.40 [0.12, 0.68] **; pre0-30: 0.66 [-0.03, 1.35]; post0-30: 0.16 [-0.23, 0.54]; post>90: 0.05 [-0.22, 0.33] |
| **Bifidobacterium** | reduced | 5 | 0.130 | 0.185 | – |
|  | full | 5 | 0.132 | 0.186 | pre45-225: -0.004 [-0.15, 0.14]; pre0-30: -0.22 [-0.64, 0.21]; post0-30: -0.20 [-0.42, 0.03]; post>90: -0.07 [-0.22, 0.09] |
| **Blautia** | reduced | 5 | 0.495 | 0.690 | – |
|  | full | 5 | 0.494 | 0.686 | pre45-225: -0.28 [-0.59, 0.02]; pre0-30: -0.32 [-0.97, 0.33]; post0-30: -0.01 [-0.40, 0.37]; post>90: 0.05 [-0.22, 0.32] |
| **Clostridium_sensu_stricto_1** | reduced | 5 | 0.079 | 0.365 | – |
|  | full | 5 | 0.093 | 0.377 | pre45-225: -0.13 [-0.51, 0.26]; pre0-30: -1.36 [-2.48, -0.24] *; post0-30: -0.03 [-0.57, 0.50]; post>90: -0.25 [-0.62, 0.12] |
| **Eggerthella** | reduced | 5 | 0.0728 | 0.379 | – |
|  | full | 5 | 0.0751 | 0.380 | pre45-225: -0.116 [-0.379, 0.147]; pre0-30: -0.233 [-0.817, 0.352]; post0-30: 0.034 [-0.334, 0.402]; post>90: -0.043 [-0.298, 0.212] |
| **Enterococcus** | reduced | 5 | 0.437 | 0.772 | – |
|  | full | 5 | 0.457 | 0.783 | pre45-225: -0.359 [-0.751, 0.033]; pre0-30: -0.917 [-1.96, 0.123]; post0-30: 1.03 [0.492, 1.56] ***; post>90: 0.531 [0.024, 1.04] * |
| **Erysipelatoclostridium** | reduced | 5 | 0.202 | 0.687 | – |
|  | full | 5 | 0.192 | 0.680 | pre45-225: 0.029 [-0.383, 0.441]; pre0-30: -0.118 [-0.906, 0.670]; post0-30: 0.301 [-0.204, 0.806]; post>90: -0.039 [-0.438, 0.359] |
| **Escherichia-Shigella** | reduced | 5 | 0.524 | 0.730 | – |
|  | full | 5 | 0.539 | 0.739 | pre45-225: -0.11 [-0.45, 0.23]; pre0-30: -1.09 [-2.13, -0.04] *; post0-30: -0.45 [-0.97, 0.07]; post>90: -0.57 [-0.96, -0.19] ** |
| **Faecalibacterium** | reduced | 5 | 0.735 | 0.823 | – |
|  | full | 5 | 0.740 | 0.825 | pre45-225: -0.63 [-0.96, -0.31] ***; pre0-30: 0.25 [-0.60, 1.11]; post0-30: -0.23 [-0.61, 0.16]; post>90: 0.03 [-0.22, 0.28] |
| **Flavonifractor** | reduced | 5 | 0.250 | 0.531 | – |
|  | full | 5 | 0.264 | 0.538 | pre45-225: 0.19 [-0.11, 0.49]; pre0-30: 0.44 [-0.11, 1.00]; post0-30: -0.13 [-0.48, 0.22]; post>90: -0.35 [-0.62, -0.09] ** |
| **Haemophilus** | reduced | 5 | 0.091 | 0.522 | – |
|  | full | 5 | 0.101 | 0.535 | pre45-225: 0.064 [-0.326, 0.454]; pre0-30: -0.756 [-1.71, 0.193]; post0-30: 0.225 [-0.287, 0.736]; post>90: 0.062 [-0.327, 0.450] |
| **Intestinibacter** | reduced | 5 | 0.138 | 0.377 | – |
|  | full | 5 | 0.140 | 0.381 | pre45-225: 0.071 [-0.215, 0.358]; pre0-30: 0.133 [-0.607, 0.872]; post0-30: 0.176 [-0.212, 0.564]; post>90: -0.059 [-0.319, 0.202] |
| **Lachnoclostridium** | reduced | 5 | 0.0723 | 0.538 | – |
|  | full | 5 | 0.0769 | 0.540 | pre45-225: -0.309 [-0.663, 0.045]; pre0-30: -0.146 [-0.829, 0.537]; post0-30: 0.256 [-0.165, 0.677]; post>90: -0.070 [-0.398, 0.257] |
| **Romboutsia** | reduced | 4 | 0.869 | 0.943 | – |
|  | full | 4 | 0.874 | 0.944 | pre45-225: -0.47 [-0.88, -0.06] *; pre0-30: -0.94 [-1.87, -0.01] *; post0-30: 0.14 [-0.30, 0.59]; post>90: -0.23 [-0.59, 0.12] |
| **Roseburia** | reduced | 5 | 0.661 | 0.836 | – |
|  | full | 5 | 0.655 | 0.835 | pre45-225: -0.14 [-0.53, 0.24]; pre0-30: 1.04 [0.24, 1.85] *; post0-30: -1.00 [-1.48, -0.53] ***; post>90: -0.02 [-0.33, 0.30] |
| **Streptococcus** | reduced | 5 | 0.079 | 0.165 | – |
|  | full | 5 | 0.092 | 0.171 | pre45-225: -0.05 [-0.38, 0.28]; pre0-30: -1.77 [-2.85, -0.68] **; post0-30: 0.25 [-0.30, 0.80]; post>90: 0.06 [-0.29, 0.40] |
| **Veillonella** | reduced | 5 | 0.507 | 0.642 | – |
|  | full | 5 | 0.517 | 0.653 | pre45-225: -0.13 [-0.49, 0.22]; pre0-30: -1.75 [-2.79, -0.71] **; post0-30: 0.15 [-0.39, 0.70]; post>90: -0.39 [-0.79, -0.002] * |

**Notes:**

- Reduced models do not include group effects (marked “–”).
- Age polynomial degree is shown; 4th-degree polynomials were used when 5th-degree models did not converge.
- CI = 95% confidence interval; *** = p<0.001, ** = p<0.01, * = p<0.05, n.s. = not significant

### Supplementary Table 4. Summary of Regression Models used in Table 2.

Zero-inflated negative binomial mixed-effects models were fitted for each taxon, with age modeled using orthogonal polynomials (5^th^ degree in reduced and full models, 7^th^ degree, in extended models), a random intercept for participant ID. Full and extended models additionally included delivery mode, duration of breastfeeding (bf.z-score), and their interactions with exposure windows (interaction indicated by colon). Only coefficients for these covariates and interactions are shown; age polynomial coefficients are not shown.

| **Taxon** | **Model** | **Age poly degree** | **Marg. R²** | **Cond. R²** | **Group effects (estimate [95% CI], significance)** |
| --- | --- | --- | --- | --- | --- |
| **Bacteroides** | Reduced | 5 | 0.111 | 0.561 | – |
|  | Full | 5 | 0.158 | 0.545 | pre45-225: 0.417 [0.050, 0.784]*; C.section: -0.413 [-0.744, -0.082]*; bf.z-score: -0.051 [-0.192, 0.090]; pre45-225:C.section: -0.199 [-0.998, 0.600]; pre45-225:bf.z-score: -0.152 [-0.527, 0.223] |
|  | Extended | 7 | 0.158 | 0.546 | pre45-225: 0.413 [0.045, 0.781]*; C.section: -0.413 [-0.744, -0.082]*; bf.z-score: -0.052 [-0.193, 0.089]; pre45-225:C.section: -0.198 [-0.997, 0.602]; pre45-225:bf.z-score: -0.149 [-0.524, 0.226] |
| **Faecalibacterium** | Reduced | 5 | 0.732 | 0.855 | – |
|  | Full | 5 | 0.750 | 0.862 | pre45-225: -1.35 [-1.79, -0.908]***; C.section: -0.130 [-0.447, 0.187]; bf.z-score: -0.184 [-0.318, -0.049]**; pre45-225:C.section: 1.57 [0.647, 2.50]**; pre45-225:bf.z-score: -0.414 [-0.895, 0.066] |
|  | Extended | 7 | 0.750 | 0.862 | pre45-225: -1.36 [-1.81, -0.917]***; C.section: -0.135 [-0.455, 0.184]; bf.z-score: -0.185 [-0.321, -0.049]**; pre45-225:C.section: 1.59 [0.657, 2.52]**; pre45-225:bf.z-score: -0.402 [-0.885, 0.082] |

**Notes:**

- Reduced models do not include group effects (marked “–”).
- CI = 95% confidence interval; *** = p<0.001, ** = p<0.01, * = p<0.05

### Supplementary Table 5. Summary of Regression Models used in Table 3.

Linear mixed-effects models were fitted for Shannon diversity or Bray–Curtis dissimilarity, with age modeled using 5th-degree orthogonal polynomials and a random intercept for participant ID. Each model additionally included a single variable of interest (delivery mode, antibiotic class, symptoms, or breast-feeding), or all variables jointly (“full model”). Only coefficients for these covariates are shown; age polynomial coefficients are not shown.

| **Dependent variable** | **Model** | **Marg. R²** | **Cond. R²** | **Group effects (estimate [95% CI], significance)** |
| --- | --- | --- | --- | --- |
| **Shannon** | Reduced (age-only) | 0.163 | 0.378 | — |
| **Shannon** | Delivery mode | 0.171 | 0.380 | Vaginal: -0.0652 [-0.319, 0.188]; C-section: 0.255 [-0.32, 0.831] |
| **Shannon** | Antibiotic class | 0.182 | 0.402 | Aminopenicillin: -0.097 [-0.479, 0.285]; Cephalosporin: 0.123 [-0.215, 0.461]; Other: -0.329 [-1.04, 0.387] |
| **Shannon** | Symptoms | 0.215 | 0.408 | ENT: 0.0684 [-0.2, 0.337]; Gastrointestinal: -1.52 [-3.02, -0.0284]*; Urogenital: 0.392 [-0.798, 1.58]; Unknown/other: -0.191 [-0.685, 0.303] |
| **Shannon** | Breast-feeding | 0.163 | 0.398 | bf.z-score: -0.0516 [-0.345, 0.242] |
| **Shannon** | Full model | 0.230 | 0.456 | Vaginal: -0.358 [-1.02, 0.309]; C-section: -0.185 [-1.11, 0.741]; bf.z-score: -0.115 [-0.435, 0.204]; ENT: 0.301 [-0.32, 0.921]; Gastrointestinal: -1.27 [-3.14, 0.61]; Urogenital: 0.789 [-0.628, 2.21]; Cephalosporin: 0.229 [-0.341, 0.8]; Other antibiotics: 0.043 [-0.907, 0.993] |
| **Bray–Curtis** | Reduced (age-only) | 0.112 | 0.465 | — |
| **Bray–Curtis** | Delivery mode | 0.130 | 0.475 | Vaginal: 0.11 [-0.168, 0.387]; C-section: -0.34 [-0.972, 0.292] |
| **Bray–Curtis** | Antibiotic class | 0.113 | 0.485 | Aminopenicillin: 0.014 [-0.413, 0.441]; Cephalosporin: 0.0196 [-0.353, 0.393]; Other: 0.201 [-0.591, 0.993] |
| **Bray–Curtis** | Symptoms | 0.182 | 0.484 | ENT: 0.132 [-0.159, 0.423]; Gastrointestinal: 1.36 [-0.245, 2.97]; Urogenital: 0.114 [-1.14, 1.37]; Unknown/other: -0.435 [-0.966, 0.095] |
| **Bray–Curtis** | Breast-feeding | 0.106 | 0.463 | bf.z-score: -0.0659 [-0.381, 0.249] |
| **Bray–Curtis** | Full model | 0.184 | 0.496 | Vaginal: -0.442 [-1.14, 0.261]; C-section: -0.95 [-1.92, 0.0232]; bf.z-score: -0.121 [-0.455, 0.213]; ENT: 0.52 [-0.131, 1.17]; Gastrointestinal: 1.79 [-0.17, 3.75]; Urogenital: 0.579 [-0.888, 2.05]; Cephalosporin: 0.258 [-0.339, 0.855]; Other antibiotics: -0.0598 [-1.05, 0.932] |

**Notes:**

- Reduced models do not include group effects (marked “–”).
- CI = 95% confidence interval; * = p<0.05, n.s. = not significant

### Supplementary Table 6. Summary of Regression Models used in Figure 6.

Results of zero-inflated negative binomial mixed models, using samples from never-treated controls and the pre45-225 group, are presented. Taxa abundances were fitted to age using orthogonal polynomials (4th- or 5th-degree, depending on model convergence), group effect (i.e. never-treated controls (ctrl) or pre45-225), and a random intercept for participant ID in reduced and full models. Full models additionally included an interaction term (denoted by colon) of group with either Shannon diversity or BC±45 in the post>90 samples of the same children. Marginal and conditional R² values, and the estimated treatment effects with corresponding confidence intervals and non-adjusted significance levels are shown; age polynomial coefficients are not displayed.

| **Taxon** | **Model** | **Degree of age polynomials** | **Marginal R²** | **Conditional R²** | **Group effects (effect size [CI], p-value)** |
| --- | --- | --- | --- | --- | --- |
| [Ruminococcus]_gnavus_group | Reduced | 5 | 0.14 | 0.673 | pre45-225: -0.283[-0.775, 0.209] |
| [Ruminococcus]_gnavus_group | Shd-full | 5 | 0.217 | 0.67 | ctrl:shd.post>90: -0.322[-0.509, -0.135]***; pre45-225:shd.post>90: -0.485[-1.04, 0.0719] |
| [Ruminococcus]_gnavus_group | BC-full | 5 | 0.236 | 0.648 | ctrl:bray.post>90: 0.369[0.192, 0.546]***; pre45-225:bray.post>90: 0.53[0.0448, 1.02]* |
|  |  |  |  |  |  |
| Actinomyces | Reduced | 5 | 0.275 | 0.572 | pre45-225: -0.186[-0.534, 0.162] |
| Actinomyces | Shd-full | 5 | 0.279 | 0.564 | ctrl:shd.post>90: 0.00852[-0.134, 0.151]; pre45-225:shd.post>90: 0.469[0.0657, 0.873]* |
| Actinomyces | BC-full | 5 | 0.278 | 0.57 | ctrl:bray.post>90: 0.0146[-0.13, 0.159]; pre45-225:bray.post>90: 0.0478[-0.354, 0.45] |
|  |  |  |  |  |  |
| Anaerostipes | Reduced | 5 | 0.698 | 0.822 | pre45-225: 0.154[-0.244, 0.551] |
| Anaerostipes | Shd-full | 5 | 0.704 | 0.824 | ctrl:shd.post>90: 0.0201[-0.105, 0.146]; pre45-225:shd.post>90: 0.392[-0.0815, 0.866] |
| Anaerostipes | BC-full | 5 | 0.694 | 0.822 | ctrl:bray.post>90: -0.0348[-0.163, 0.0936]; pre45-225:bray.post>90: -0.0227[-0.471, 0.426] |
|  |  |  |  |  |  |
| Bacteroides | Reduced | 5 | 0.116 | 0.484 | pre45-225: 0.466[0.16, 0.772]** |
| Bacteroides | Shd-full | 5 | 0.131 | 0.507 | ctrl:shd.post>90: 0.0474[-0.0736, 0.168]; pre45-225:shd.post>90: 0.652[0.302, 1]*** |
| Bacteroides | BC-full | 5 | 0.106 | 0.509 | ctrl:bray.post>90: -0.0527[-0.177, 0.0718]; pre45-225:bray.post>90: 0.0581[-0.273, 0.389] |
|  |  |  |  |  |  |
| Bifidobacterium | Reduced | 5 | 0.12 | 0.186 | pre45-225: -0.0388[-0.204, 0.126] |
| Bifidobacterium | Shd-full | 5 | 0.127 | 0.188 | ctrl:shd.post>90: -0.0606[-0.122, 0.000741]; pre45-225:shd.post>90: -0.149[-0.334, 0.0367] |
| Bifidobacterium | BC-full | 5 | 0.128 | 0.184 | ctrl:bray.post>90: -0.119[-0.179, -0.0587]***; pre45-225:bray.post>90: 0.0119[-0.16, 0.184] |
|  |  |  |  |  |  |
| Blautia | Reduced | 5 | 0.472 | 0.749 | pre45-225: -0.628[-1.02, -0.24]** |
| Blautia | Shd-full | 5 | 0.481 | 0.735 | ctrl:shd.post>90: 0.0382[-0.0968, 0.173]; pre45-225:shd.post>90: 0.716[0.322, 1.11]*** |
| Blautia | BC-full | 5 | 0.489 | 0.739 | ctrl:bray.post>90: 0.161[0.0272, 0.294]*; pre45-225:bray.post>90: -0.388[-0.776, 0.00014] |
|  |  |  |  |  |  |
| Clostridium_sensu_stricto_1 | Reduced | 5 | 0.0797 | 0.33 | pre45-225: -0.209[-0.626, 0.207] |
| Clostridium_sensu_stricto_1 | Shd-full | 5 | 0.0862 | 0.321 | ctrl:shd.post>90: 0.0573[-0.0763, 0.191]; pre45-225:shd.post>90: -0.219[-0.773, 0.335] |
| Clostridium_sensu_stricto_1 | BC-full | 5 | 0.092 | 0.319 | ctrl:bray.post>90: 0.109[-0.0231, 0.241]; pre45-225:bray.post>90: -0.112[-0.578, 0.355] |
|  |  |  |  |  |  |
| Eggerthella | Reduced | 5 | 0.109 | 0.387 | pre45-225: -0.123[-0.408, 0.163] |
| Eggerthella | Shd-full | 5 | 0.125 | 0.379 | ctrl:shd.post>90: -0.116[-0.223, -0.00867]*; pre45-225:shd.post>90: 0.117[-0.208, 0.442] |
| Eggerthella | BC-full | 5 | 0.127 | 0.382 | ctrl:bray.post>90: 0.103[-0.00439, 0.21]; pre45-225:bray.post>90: 0.0787[-0.24, 0.397] |
|  |  |  |  |  |  |
| Enterococcus | Reduced | 5 | 0.5 | 0.744 | pre45-225: -0.514[-0.884, -0.144]** |
| Enterococcus | Shd-full | 5 | 0.524 | 0.743 | ctrl:shd.post>90: -0.0874[-0.231, 0.0558]; pre45-225:shd.post>90: -0.583[-1.02, -0.141]* |
| Enterococcus | BC-full | 5 | 0.514 | 0.75 | ctrl:bray.post>90: -0.0636[-0.209, 0.0817]; pre45-225:bray.post>90: -0.517[-0.949, -0.0857]* |
|  |  |  |  |  |  |
| Erysipelatoclostridium | Reduced | 5 | 0.188 | 0.726 | pre45-225: -0.327[-0.808, 0.153] |
| Erysipelatoclostridium | Shd-full | 5 | 0.233 | 0.729 | ctrl:shd.post>90: -0.264[-0.452, -0.0747]**; pre45-225:shd.post>90: 0.397[-0.145, 0.939] |
| Erysipelatoclostridium | BC-full | 5 | 0.267 | 0.716 | ctrl:bray.post>90: 0.358[0.175, 0.54]***; pre45-225:bray.post>90: 0.126[-0.365, 0.617] |
|  |  |  |  |  |  |
| Escherichia-Shigella | Reduced | 5 | 0.519 | 0.723 | pre45-225: -0.256[-0.617, 0.106] |
| Escherichia-Shigella | Shd-full | 5 | 0.524 | 0.72 | ctrl:shd.post>90: -0.0982[-0.242, 0.0456]; pre45-225:shd.post>90: 0.194[-0.221, 0.609] |
| Escherichia-Shigella | BC-full | 5 | 0.518 | 0.721 | ctrl:bray.post>90: -0.0653[-0.211, 0.0801]; pre45-225:bray.post>90: -0.363[-0.747, 0.0214] |
|  |  |  |  |  |  |
| Faecalibacterium | Reduced | 5 | 0.739 | 0.867 | pre45-225: -1.4[-1.81, -0.976]*** |
| Faecalibacterium | Shd-full | 5 | 0.736 | 0.832 | ctrl:shd.post>90: 0.192[0.0776, 0.306]**; pre45-225:shd.post>90: 0.475[0.062, 0.888]* |
| Faecalibacterium | BC-full | 5 | 0.727 | 0.833 | ctrl:bray.post>90: -0.139[-0.257, -0.0206]*; pre45-225:bray.post>90: -0.203[-0.627, 0.222] |
|  |  |  |  |  |  |
| Flavonifractor | Reduced | 5 | 0.225 | 0.58 | pre45-225: -0.00201[-0.363, 0.359] |
| Flavonifractor | Shd-full | 5 | 0.262 | 0.569 | ctrl:shd.post>90: -0.121[-0.241, -0.00188]*; pre45-225:shd.post>90: -0.39[-0.791, 0.012] |
| Flavonifractor | BC-full | 5 | 0.262 | 0.573 | ctrl:bray.post>90: 0.144[0.0208, 0.268]*; pre45-225:bray.post>90: 0.465[0.0656, 0.864]* |
|  |  |  |  |  |  |
| Haemophilus | Reduced | 5 | 0.0939 | 0.52 | pre45-225: 0.0805[-0.347, 0.508] |
| Haemophilus | Shd-full | 5 | 0.0996 | 0.522 | ctrl:shd.post>90: -0.0519[-0.219, 0.115]; pre45-225:shd.post>90: 0.317[-0.166, 0.8] |
| Haemophilus | BC-full | 5 | 0.107 | 0.525 | ctrl:bray.post>90: 0.121[-0.0475, 0.289]; pre45-225:bray.post>90: -0.242[-0.68, 0.195] |
|  |  |  |  |  |  |
| Intestinibacter | Reduced | 5 | 0.159 | 0.412 | pre45-225: 0.0917[-0.22, 0.403] |
| Intestinibacter | Shd-full | 5 | 0.161 | 0.409 | ctrl:shd.post>90: 0.0626[-0.0441, 0.169]; pre45-225:shd.post>90: 0.0314[-0.356, 0.418] |
| Intestinibacter | BC-full | 5 | 0.181 | 0.424 | ctrl:bray.post>90: 0.0694[-0.0368, 0.176]; pre45-225:bray.post>90: 0.322[-0.0248, 0.668] |
|  |  |  |  |  |  |
| Lachnoclostridium | Reduced | 5 | 0.0679 | 0.579 | pre45-225: -0.47[-0.882, -0.0574]* |
| Lachnoclostridium | Shd-full | 5 | 0.0864 | 0.574 | ctrl:shd.post>90: -0.162[-0.32, -0.0039]*; pre45-225:shd.post>90: 0.0939[-0.377, 0.565] |
| Lachnoclostridium | BC-full | 5 | 0.109 | 0.575 | ctrl:bray.post>90: 0.23[0.076, 0.384]**; pre45-225:bray.post>90: -0.228[-0.633, 0.177] |
|  |  |  |  |  |  |
| Romboutsia | Reduced | 4 | 0.887 | 0.953 | pre45-225: -0.759[-1.23, -0.284]** |
| Romboutsia | Shd-full | 4 | 0.88 | 0.95 | ctrl:shd.post>90: 0.0692[-0.101, 0.24]; pre45-225:shd.post>90: 0.172[-0.373, 0.717] |
| Romboutsia | BC-full | 4 | 0.883 | 0.951 | ctrl:bray.post>90: 0.0336[-0.134, 0.201]; pre45-225:bray.post>90: -0.369[-0.864, 0.127] |
|  |  |  |  |  |  |
| Roseburia | Reduced | 5 | 0.67 | 0.86 | pre45-225: -0.222[-0.696, 0.251] |
| Roseburia | Shd-full | 5 | 0.675 | 0.85 | ctrl:shd.post>90: 0.176[0.0254, 0.326]*; pre45-225:shd.post>90: 0.609[0.0844, 1.13]* |
| Roseburia | BC-full | 5 | 0.672 | 0.858 | ctrl:bray.post>90: 0.012[-0.142, 0.166]; pre45-225:bray.post>90: -0.517[-1.06, 0.0238] |
|  |  |  |  |  |  |
| Streptococcus | Reduced | 5 | 0.0783 | 0.145 | pre45-225: -0.0842[-0.44, 0.271] |
| Streptococcus | Shd-full | 5 | 0.0792 | 0.143 | ctrl:shd.post>90: -0.0295[-0.135, 0.0758]; pre45-225:shd.post>90: 0.0437[-0.37, 0.458] |
| Streptococcus | BC-full | 5 | 0.0812 | 0.151 | ctrl:bray.post>90: 0.0384[-0.0687, 0.145]; pre45-225:bray.post>90: 0.176[-0.209, 0.561] |
|  |  |  |  |  |  |
| Veillonella | Reduced | 5 | 0.472 | 0.616 | pre45-225: -0.169[-0.548, 0.21] |
| Veillonella | Shd-full | 5 | 0.511 | 0.626 | ctrl:shd.post>90: -0.241[-0.368, -0.114]***; pre45-225:shd.post>90: 0.027[-0.395, 0.449] |
| Veillonella | BC-full | 5 | 0.5 | 0.618 | ctrl:bray.post>90: 0.172[0.0421, 0.301]*; pre45-225:bray.post>90: -0.0183[-0.412, 0.375] |

**Notes:**

- Age polynomial degree is shown; 4th-degree polynomials were used when 5th-degree models did not converge.
- CI = 95% confidence interval; *** = p<0.001, ** = p<0.01, * = p<0.05

### Supplementary Table 7. Summary of additional Regression Models related to Figure 6.

Regression models for the abundance of *Bacteroides* or *Blautia* in the never-treated control group were fitted using zero-inflated negative binomial mixed models. Age was modeled as a 5th-degree orthogonal polynomial and included as a fixed effect (coefficients not shown), with participant ID as a random factor. Full models additionally included interactions (denoted by colon) between age intervals (time frames as indicated) and Shannon diversity in the post>90 samples of the same children. Marginal and conditional R² values, and the estimated treatment effects with corresponding confidence intervals and non-adjusted significance levels are shown; age polynomial coefficients are not displayed.

| **Taxon** | **Model** | **Marginal R²** | **Conditional R²** | **Group effects (effect size [CI], p-value)** |
| --- | --- | --- | --- | --- |
| Bacteroides | reduced | 0.131 | 0.513 | — |
| Bacteroides | full | 0.143 | 0.519 | cut.age[3,215]:shd.post>90: -0.0804 [-0.257, 0.0957]; cut.age(215,427]:shd.post>90: 0.0457 [-0.13, 0.221]; cut.age(427,639]:shd.post>90: 0.156 [-0.0115, 0.324]; cut.age(639,851]:shd.post>90: 0.155 [-0.0604, 0.371] |
| Blautia | reduced | 0.479 | 0.706 | — |
| Blautia | full | 0.479 | 0.707 | cut.age[3,215]:shd.post>90: 0.273 [0.0575, 0.489]*; cut.age(215,427]:shd.post>90: 0.0594 [-0.109, 0.228]; cut.age(427,639]:shd.post>90: 0.106 [-0.0469, 0.258]; cut.age(639,851]:shd.post>90: 0.0387 [-0.159, 0.237] |

**Notes:**

- Reduced models do not include group effects (marked “–”).
- CI = 95% confidence interval; * = p<0.05

### Supplementary Table 8. Summary of Regression Models used in Figure 7.

Data were stratified into samples from children with generally high or low abundances of *Bacteroides* or *Blautia*, respsectively. Linear mixed-effects regression models were fitted within each stratum to either Shannon diversity or Bray–Curtis dissimilarity (BC), with age modeled as a 5th-degree orthogonal polynomial (coefficients not shown) and participant ID included as a random factor. Group effects are reported for pre45-225, pre0-30, post0-30, and post>90 including estimates, confidence intervals, and significance levels. Marginal and conditional R² could not be calculated due to missing data in the pre0-30 group.

| **DV** | **Strata** | **Group effects (effect size [CI], p-value)** |
| --- | --- | --- |
| Shannon | Bacteroides low | pre45-225: 0.0081 [-0.214, 0.23]; post0-30: -0.415 [-0.727, -0.102]**; post>90: -0.203 [-0.445, 0.039] |
| Shannon | Bacteroides high | pre45-225: 0.112 [-0.064, 0.288]; pre0-30: 0.417 [0.04, 0.795]*; post0-30: -0.0331 [-0.319, 0.252]; post>90: 0.175 [-0.00137, 0.351] |
| Shannon | Blautia low | pre45-225: 0.0168 [-0.203, 0.237]; pre0-30: 0.0173 [-0.727, 0.762]; post0-30: -0.293 [-0.732, 0.147]; post>90: 0.0701 [-0.16, 0.301] |
| Shannon | Blautia high | pre45-225: 0.0265 [-0.152, 0.205]; pre0-30: 0.495 [0.0431, 0.946]**; post0-30: -0.236 [-0.469, -0.00247]**; post>90: -0.0568 [-0.243, 0.13] |
| BC | Bacteroides low | pre45-225: -0.00833 [-0.0518, 0.0351]; post0-30: 0.0649 [0.00533, 0.125]*; post>90: 0.000829 [-0.0461, 0.0478] |
| BC | Bacteroides high | pre45-225: -0.00404 [-0.0379, 0.0299]; pre0-30: 0.0272 [-0.0441, 0.0984]; post0-30: 0.000211 [-0.0539, 0.0543]; post>90: 0.00903 [-0.025, 0.043] |
| BC | Blautia low | pre45-225: -0.00241 [-0.041, 0.0362]; pre0-30: 0.0286 [-0.0994, 0.157]; post0-30: 0.0209 [-0.0548, 0.0967]; post>90: 0.0175 [-0.0229, 0.0579] |
| BC | Blautia high | pre45-225: -0.0191 [-0.0552, 0.0169]; pre0-30: 0.0197 [-0.0721, 0.111]; post0-30: 0.0339 [-0.0133, 0.0812]; post>90: -0.00443 [-0.0421, 0.0333] |

**Notes:**

- DV = dependent variable
- CI = 95% confidence interval; ** = p<0.01, * = p<0.05, n.s. = not significant

Supplementary Data

**Supplementary Data 1. Metadata.**

The Excel table shows the metadata for all participants and samples.
